# Effectiveness of bridge-in, objective, pre-assessment, participatory learning, post-assessment, and summary teaching strategy in Chinese medical education: A systematic review and meta-analysis

**DOI:** 10.3389/fmed.2022.975229

**Published:** 2022-09-15

**Authors:** Xingming Ma, Dequan Zeng, Jie Wang, Kun Xu, Ling Li

**Affiliations:** ^1^School of Health Management, Xihua University, Chengdu, China; ^2^Health Promotion Center, Xihua University, Chengdu, China; ^3^School of Food and Biological Engineering, Xihua University, Chengdu, China

**Keywords:** BOPPPS (bridge-in, objective, pre-assessment, participatory learning, post-assessment, and summary), clinical medicine, medical education, medical students, curriculum, traditional learning, meta-analysis

## Abstract

The BOPPPS teaching strategy has been used recently in many medical courses as an improved and more practical pedagogy in China. Nevertheless, the effectiveness of this pedagogy has not been fully assessed in terms of knowledge and skill outcomes in medical education. This meta-analysis aimed to evaluate the effectiveness of the BOPPPS strategy compared with traditional lecture-based learning (LBL) in Chinese medical education. The English electronic databases of Web of Science, PubMed, Embase, and the Cochrane Library and the Chinese electronic databases of CNKI, CQVIP, Wanfang, and CBM were used to search the publications related to the BOPPPS teaching strategy before 6 Jun 2022. Eligibility publications were retrieved and the data were extracted by two researchers independently according to the predefined inclusion and exclusion criteria. Quality analysis was performed using the Cochrane risk-of-bias tool, and the meta-analysis was performed using RevMan 5.3 and StataSE. We retrieved 367 records and 41 studies with a total of 5,042 medical students in the meta-analysis, which included 34 randomized controlled trials (RCTs) and 7 Cohort studies. In the cumulative meta-analysis, BOPPPS strategy significantly increased skill scores (SS) (SMD: 1.15, 95% CI: 1.00–1.30, *P* < 0.00001), knowledge examination scores (KES) (SMD: 1.56, 95% CI: 1.24–1.89, *P* < 0.00001), comprehensive ability scores (CAS) (SMD: 1.22, 95%CI: 0.85–1.59; *P* < 0.00001), and teaching satisfaction (TS) (OR: 3.64; 95%CI: 2.97–4.46; *P* < 0.0001) compared to the LBL model among those medical students. Statistically similar results were obtained in the sensitivity analysis. These results showed that the BOPPPS method is an effective teaching strategy for Chinese medical students to improve SS, knowledge scores, CAS, and TS when compared with LBL in medical education. Because of the limited quantity and quality of the included studies, further rigorous studies are needed to conclude with more confidence.

## Introduction

The BOPPPS teaching strategy was first proposed by Douglas Kerrin from the University of British Columbia in 1978 (1), which divides into six distinct steps: bridge-in (B), objective (O), pre-assessment (P), participatory learning (P), post-assessment (P), and summary (S), i.e., BOPPPS. Based on the constructivism learning theory, the BOPPPS teaching strategy constructs a complete framework and process for the achievement of teaching objectives (2). The flowchart of class design for the BOPPPS strategy compared to lecture-based learning (LBL) was attached in Supplementary Appendix 1.

In Chinese universities, the BOPPPS teaching strategy was firstly attempted in 2011 for the improvement of teaching effectiveness and comprehensive ability in learning plant ecology for postgraduates (3), classroom teaching quality of English courses (4), and academic performance of medical cell biology course for graduates compared with the traditional instructions (5). Recently, it has been developed and extensively trialed in China’s higher medical education to increase the efficiency of education and learning processes due to a clear organizing teaching process and to convert students into active learners (6, 7).

Traditionally, the instructor is the center of the class teaching with a full lecture in the classroom, which may inhibit the learning initiative and enthusiasm of students, and consequently, their interaction and active learning are limited with the passive receiving the knowledge (7, 8). For the BOPPPS teaching strategy, it forms a systematic, coherent, and operational six-step of teaching, which could help instructors in disassembling and analyzing teaching activities to improve the teaching process (8). According to the closed-loop teaching six-step, the instructional content could be conveniently designed, and the instructional process could be evaluated and revised by instructors. The BOPPPS teaching strategy further focuses on the participation of students, interaction, and feedback ability and strengthens the closed-loop teaching unit. Compared with LBL, the core objects of the BOPPPS strategy is a learner-centered teaching activity in which learners participate in discussing the content with peers or mentors during class, self-assessment of learning effects, and objectives after class (9). Particularly during the participatory learning phase, students can participate in various active learning activities such as problem-discussion and immediate feedback which emphasizes full interaction between classmates and/or teachers to stimulate Students’ enthusiasm and interest for the improvement of self-efficacy, knowledge, and communication ability (2).

For the past 10 years, the BOPPPS teaching strategy has been used in teaching many medical subjects, covering nearly all medical disciplines such as internal medicine, surgery, pathology, physiology, healthcare, management education, etc. in China (10). There has been a beneficial shift in academic accomplishment levels in medical disciplines education, with an increase in interest in the BOPPPS teaching strategy. Increased research has been published on BOPPPS in Chinese medical education over the last decade. Various studies on the effectiveness of BOPPPS in medical education have been conducted. These studies reported positive effects on the development of Student’s skills and knowledge, and the improvement of self-learning ability, academic performance, and learning satisfaction, as compared to the control group that received traditional LBL instruction.

Nevertheless, the outcomes of the BOPPPS teaching strategy on medical education have been ambiguous or inconsistent in China. Most studies have a small sample size, and few have used quantitative synthesis in the evaluation of the effectiveness of the BOPPPS teaching strategy. It is possible and valuable to conduct a quantitative synthesis of the data using rigorous methods. This meta-analysis aimed to evaluate the overall effectiveness of the BOPPPS teaching strategy compared to LBL teaching in China’s higher medical education, which included final knowledge examination score (KES), skill score (SS), comprehensive ability score (CAS), and teaching satisfaction (TS).

## Materials and methods

### Study design

This meta-analysis and systematic review were designed according to the PRISMA (preferred reporting items for systematic reviews and meta-analyses, PRISMA) guidelines (11). The checklist included items deemed essential for transparent reporting of a systematic review. The PRISMA Checklist was attached in Supplementary Appendix 2.

### Selection criteria

The PICOS (population, intervention, comparison, outcome, and study design) framework was used to determine the inclusion criteria of studies. The following studies will be included: (a) the participants for the studies were medical students in Chinese medical schools; (b) the experimental group received the intervention of BOPPPS teaching strategy; (c) the groups of LBL were as control; (d) the core curriculums covered clinical medicine and/or biomedicine disciplines; (e) the studies were two-group controlled (randomized/non-randomized); (f) the outcomes presented as data or descriptions of each controlled studies included at least one of the following measurements: SS, KES, CAS, TS; (g) only studies full-text published in English language and Chinese language were included; (f) all above-mentioned studies conducted before 6 Jun 2022.

### Exclusion criteria

Any study which did not meet the aforementioned inclusion criteria was excluded. The following studies will be excluded: (a) the participants of non-clinical medicine students (e.g., dental, nursing, pharmacy, or any allied health profession) in Chinese medical schools; (b) incomplete the six-stage of BOPPPS teaching strategy in the experimental group; (c) reviews, editorials, conference papers, case reports; (d) duplicate studies or overlapping participants; (e) insufficient data to calculate the outcomes and/or not report a quantitative outcome for the effectiveness; (f) non-English language and non-Chinese language studies.

### Search strategy

PubMed, Web of Science, Embase, and Cochrane Library were searched without any restrictions before 6 Jun 2022. Asterisks were used as a truncation symbol for searching. English key search terms included BOPPPS (bridge, objective, pre-assessment, participatory, post-assessment, summary) and student*. Chinese electronic databases of CNKI, Wanfang, CQVIP, and CBM were searched before 6 Jun 2022. Chinese key search terms included BOPPPS and medicine and student. The specific set of MeSH terms or title or keywords or abstract was used to search the English databases, and the topic or title or keywords or abstract was used to search the Chinese databases. The retrieved strategy of studies from databases was shown in Supplementary Appendix 3.

### Data extraction

According to a predefined form, data were searched, collected, and extracted by two independent reviewers (K. Xu and J. Wang). From each study, the following information was extracted: the surname of the first author, year of publication, student characteristics and sample size of the participants, course name, intervention methods, and outcome measures. Any disagreements between these two reviewers on the eligibility of the extracted data were resolved through discussion with a third research team member (X. Ma) until an agreement was reached.

### Outcomes

In this meta-analysis, the primary outcomes were the final KES, SS, and CAS in the BOPPPS group and the control group. The TS was a secondary outcome. Subgroup analyses of study design, hometown, training levels (undergraduates and graduates), course type (theory and practice), and course contents were conducted to find out a potential source of heterogeneity in the above outcomes.

### Risk of bias assessment

The Cochrane risk of bias 2 (RoB2 v9) tool was used to evaluate the quality of individual included studies (12). Evaluation criteria included the following five domains and overall bias: randomization process, deviation from intended interventions, missing outcome data, measurement of the outcome, and selection of the reported result. Funnel plots were used to evaluate publication bias. During the assessment process, two reviewers (K. Xu and J. Wang) independently conducted the risk of bias. Disagreements would be resolved through consensus or the involvement of a third reviewer (D. Zeng). According to the Cochrane risk of bias instructions, the risk of bias for each study was rated as high, with some concerns, and low risk. For any single outcome, studies with a high risk of overall bias were excluded from the meta-analysis.

### Statistical analysis

Statistical analysis of heterogeneity and the meta-analysis for effectiveness outcomes were performed using the Review Manager (RevMan) software (V.5.3, the Cochrane Collaboration, UK). Galbraith plot analysis of heterogeneity was carried out using the StataSE software (version 14, StataCorp LP USA). For the continuous data including SS, KES, and CAS, the effect sizes were estimated with the standardized mean difference (SMD) and its 95% confidence intervals (CI). The odds ratio (OR) and 95%CI were calculated to estimate the dichotomous data such as TS. Publication bias was examined independently in funnel plots (13). The *Z*-test was used for overall effect and the Chi-square test for multiple subgroups comparison was performed to evaluate the subgroup’s result differences, and *P* < 0.05 was considered statistically significant (14). Heterogeneity among included studies was evaluated with the inconsistency (*I*^2^) metric (15). Degrees of statistical heterogeneity were low (*I*^2^<30%), moderate (*I*^2^ = 30–50%), and high (*I*^2^>50%), respectively. If *I*^2^ was < 50%, the fixed-effects model was used, which represents low to moderate heterogeneity or no statistical heterogeneity among the studies. Otherwise, the random effect model was used for analysis (*I*^2^ ≥ 50%) (16).

## Results

### Database searching and selection

**FIGURE 1:**
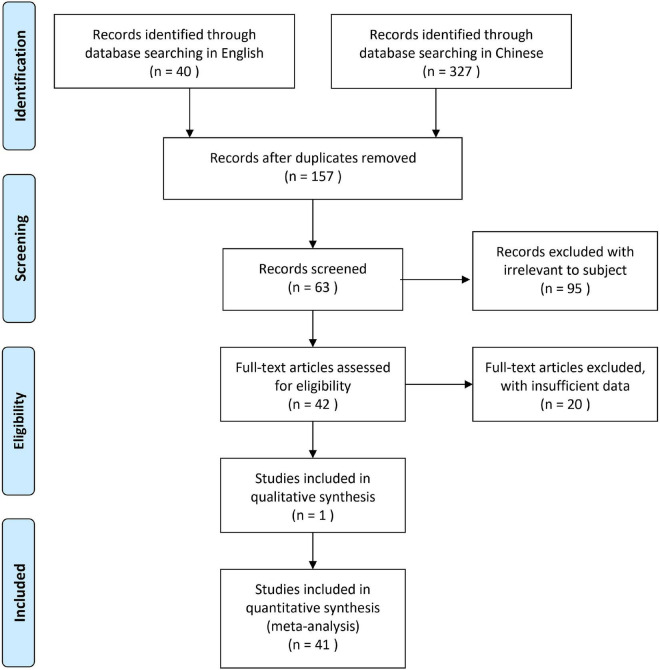
The methodological flowchart of PRISMA for the selection of the included studies in this meta-analysis.

The methodological flowchart of PRISMA was shown in Figure 1. A total of 367 potentially relevant records were firstly retrieved from the electronic database, and a total of 210 duplicate records were excluded. According to the title and abstract, 95 publications were then excluded because some were irrelevant to the subject of the meta-analysis such as nursing, stomatology, pharmacy, and others were experience summaries or questionnaire surveys without quantitative measurement of the scores. After reading the full text, another 20 articles were excluded since some were insufficient data to be extracted (*n* = 14), and/or trials without controls (*n* = 6). Eventually, 41 RCTs were included in this meta-analysis based on the inclusion criteria.

### Study characteristics

The basic characteristics of the 41 studies were presented in Table 1. All of the studies that were included concerning medical curriculum education for students of MBBS (Bachelor of medicine and bachelor of surgery). The years of the publications were before 6 Jun 2022. A total of 2,305 medical students participated in the BOPPPS teaching strategy and 2,737 medical students in the LBL, which were included in the 41 studies (17–57). All of the students in included studies were from medical school. Out of these 41 articles, only one was published in English, and forty were published in Chinese. There were 34 randomized controlled trials (RCTs), and 7 Cohort studies compared with the previous year’s Students’ scores.

**TABLE 1:** Main characteristics of the included studies in the current meta-analysis.

References	Study design	Sample size (BOPPPS)	Sample size (LBL)	Population*	Course name	Course type	Outcome measures	RoB2
Chen et al. (17)	RCT	34	34	Graduates	TCM	Practice	KES, CAS, TS	L
Chen et al. (18)	RCT	100	100	Undergraduates	Pathology	Theory	KES, CAS	L
Cheng et al. (19)	RCT	36	32	Undergraduates	Diagnostics (physical diagnostics)	Practice	KES, CAS	L
Chu et al. (20)	RCT	87	89	Undergraduates	Human anatomy	Theory	KES, CAS	L
Deng et al. (21)	CS	57	55	Undergraduates	General medicine	Theory	KES, TS	L
Di et al. (22)	RCT	58	58	Undergraduates	Internal medicine (respiratory)	Practice	KES, CAS	S
Duan et al. (23)	RCT	55	52	Undergraduates	Surgery (orthopedics)	Theory	KES, SS, CAS, TS	L
Gao et al. (24)	RCT	75	78	Undergraduates	Internal medicine	Practice	KES, TS	L
Gu and Ji (25)	RCT	30	30	Graduates	Surgery (neurosurgery)	Practice	KES, CAS, TS	L
Guo (26)	RCT	118	120	Undergraduates	Emergency medicine	Theory	KES, TS	L
Li et al. (27)	RCT	16	16	Graduates	Diagnostics (radiodiagnostics)	Practice	KES, CAS, TS	L
Li et al. (28)	RCT	108	109	Undergraduates	Obstetrics and gynecology	Practice	CAS, TS	L
Li et al. (29)	RCT	20	20	Undergraduates	Internal medicine	Practice	KES, CAS	S
Li et al. (30)	CS	81	74	Undergraduates	Diagnostics (clinical diagnostics)	Theory	KES, SS, CAS	L
Li et al. (31)	CS	57	28	Undergraduates	TCM	Theory	KES	L
Li et al. (32)	RCT	66	69	Undergraduates	Pediatrics	Practice	CAS	S
Liu et al. (33)	RCT	30	30	Undergraduates	TCM	Practice	CAS, TS	L
Liu et al. (34)	CS	518	532	Undergraduates	Physiology	Theory	KES	S
Liu et al. (35)	RCT	36	36	Undergraduates	Surgery (spine)	Theory	KES	L
Ma et al. (36)	RCT	25	26	Undergraduates	Evidence-based medicine	Theory	KES, TS	L
Miao et al. (37)	RCT	28	28	Graduates	Anesthesiology	Practice	KES, SS, CAS, TS	L
Parhati et al. (38)	RCT	32	32	Undergraduates	Surgery (orthopedics)	Theory	KES, SS, TS	L
Qin et al. (39)	RCT	15	15	Undergraduates	Internal medicine (neurology)	Practice	KES, SS	L
Shen et al. (40)	RCT	32	34	Undergraduates	Diagnostics (clinical diagnostics)	Theory	KES, TS	L
Shen et al. (41)	CS	68	66	Undergraduates	Internal medicine	Theory	KES, TS	L
Sun et al. (42)	CS	55	55	Undergraduates	Pathology	Theory	KES	L
Tan et al. (43)	RCT	28	28	Undergraduates	Pediatrics	Practice	KES, CAS	L
Tao and Wang (44)	RCT	52	52	Graduates	Surgery (general surgery)	Practice	KES, SS, TS	L
Wan et al. (45)	RCT	32	32	Undergraduates	Anesthesiology	Theory	KES, TS	L
Wang et al. (46)	RCT	39	35	Undergraduates	Internal medicine (neurology)	Practice	KES, TS	L
Xing (47)	RCT	43	43	Undergraduates	Internal medicine (oncology)	Practice	KES, CAS, TS	L
Xu et al. (48)	CS	98	63	Undergraduates	TCM	Theory	KES	L
Yang and Meng (49)	RCT	116	119	Undergraduates	Obstetrics and gynecology	Theory	KES, TS	L
Yang et al. (50)	RCT	51	48	Undergraduates	Human anatomy	Theory	CAS	L
Yao and Ying (51)	RCT	30	30	Undergraduates	TCM	Practice	KES, SS, CAS, TS	L
Zhang et al. (52)	RCT	30	30	Undergraduates	Obstetrics and gynecology	Theory	KES, TS	L
Zhang et al. (53)	RCT	30	35	Graduates	Obstetrics and gynecology	Practice	KES, SS	L
Zhao et al. (54)	RCT	240	214	Graduates	Internal medicine (respiratory)	Practice	KES, CAS, TS	L
Zhao et al. (55)	RCT	115	112	Undergraduates	Surgery	Practice	KES, SS, TS	L
Zhou et al. (56)	RCT	30	30	Undergraduates	Internal medicine (urology)	Theory	KES	L
Zhou and Fu (57)	RCT	52	48	Undergraduates	Diagnostics (electrocardiogram)	Theory	KES, SS	L

CS, Cohort study and compared with previous year Students’ score; RCT, randomized controlled trial; BOPPPS, bridge-in, objective, pre-assessment, participatory learning, post-assessment, and summary; LBL, lecture-based learning.

*Graduates, residents with standardized clinical training; Undergraduates, medical students from freshmen to five-grade in the school.

TCM, traditional Chinese medicine; KES, knowledge examination score; CAS, comprehensive ability score; SS, skill score; TS, teaching satisfaction.

RoB2, The Cochrane risk of bias 2; H, overall bias of high; L, overall bias of low; and S, overall bias of some concerns.

There were 21 trials on theoretical courses and 20 trials on practical courses. There were 34 trials related to undergraduates of clinical medicine and 7 trials on university graduates with standardized clinical training in hospitals. In addition, the curriculums for medical students included 5 studies for basic medicine courses, 5 studies for traditional Chinese medicine (TCM) courses, and 31 studies for clinical medicine courses (Table 1). The trial curriculums in the included studies had 9 studies on the knowledge of internal medicine (6, 8, 13, 23, 25, 30, 31, 38), 6 studies on surgery (7, 9, 19, 22, 28, 39), 5 studies on TCM (1, 15, 17, 32, 35), 5 studies on clinical diagnostics (3, 11, 14, 24, 41), 4 studies on obstetrics and gynecology (12, 33, 36, 37), 2 studies on anesthesiology (24, 33), 2 studies on human anatomy (4, 34), 2 studies on pathology (2, 26), 2 studies on pediatrics (16, 27), 1 studies on emergency medicine (10), 1 studies on evidence-based medicine (20), 1 studies on general medicine (5), and 1 studies on physiology (18).

### Quality assessment

**FIGURE 2:**
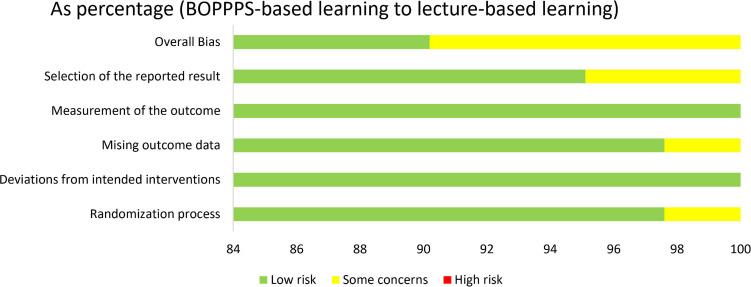
Risk of bias of included RCTs with the Cochrane RoB2 tool.

**FIGURE 3:**
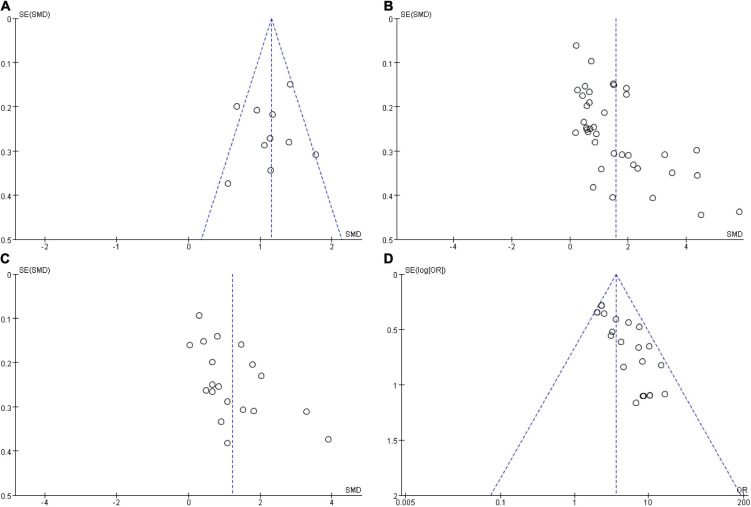
Funnel plots for publication bias. **(A)** Publication bias of skill scores; **(B)** publication bias of knowledge examination scores; **(C)** publication bias of comprehensive ability scores; **(D)** publication bias of teaching effect satisfactory. OR, odds ratio; SMD, standardized mean difference.

According to the instructions in the Cochrane Collaboration Handbook (12), the assessment for each outcome included the five domain ratings plus the overall judgment. According to five domain ratings, the overall bias of each included study was considered as “low risk of bias.” All the included studies reported their outcome data and therefore the majority (*n* = 37, 90.2%) had low attrition bias, four studies (9.8%) had some unclear risk of bias due to insufficient information on allocation and outcome data, and the majority of included studies were of high quality in Figure 2 for overall assessment and in Supplementary Appendix 4 for each included study. Then, the shape of the funnel plot of KES, SS, CAS, and TS was nearly symmetrical, indicating negligible evidence of significant publication bias (Figures 3A–D).

### Evaluation of the effectiveness of bridge-in, objective, pre-assessment, participatory learning, post-assessment, and summary compared with lecture-based learning

#### Measurements of skill scores

**FIGURE 4:**
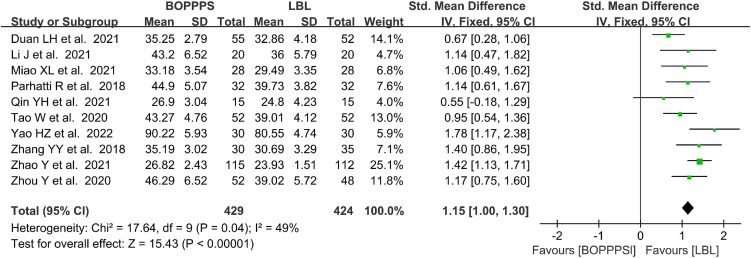
Forest plot of skill scores for BOPPPS compared with LBL.

There were 10 included studies data related to the SS evaluation with 429 and 424 students in the BOPPPS and LBL groups, respectively. Compared with the LBL teaching, the pooled effect of 10 studies (SMD 1.15, 95% CI: 1.00–1.30, *Z* = 15.43, *P* < 0.00001) showed a significant improving effect on SS in the group of BOPPPS. The fixed-effects model was used for the meta-analysis because of the moderate heterogeneity (*P* = 0.04, *I*^2^ = 49% < 50%) of the data (Figure 4).

#### Measurements of final knowledge examination score

**FIGURE 5:**
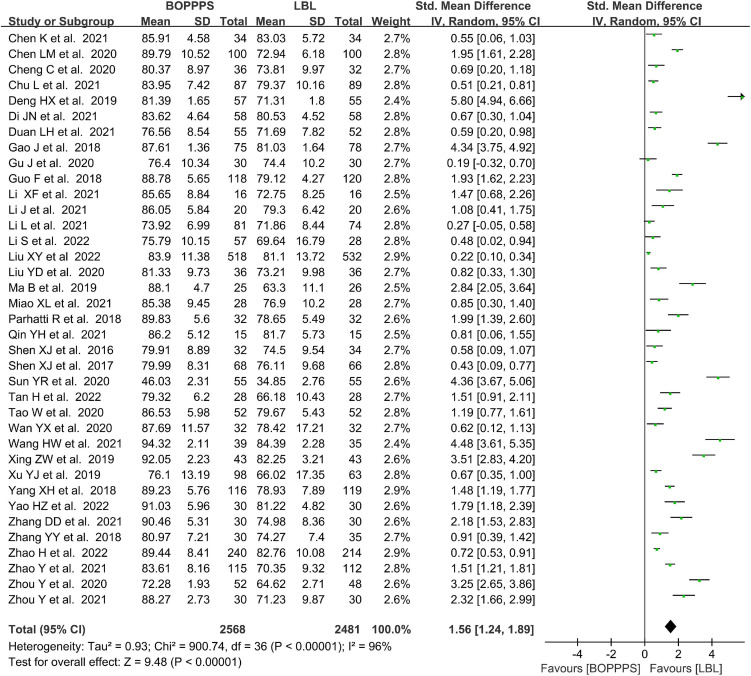
Forest plot of knowledge examination scores for BOPPPS compared with LBL.

There were 37 included studies data related to the final KES evaluation with 2,568 and 2,481 students in the BOPPPS and LBL groups, respectively. The pooled effect of those studies (SMD 1.56, 95% CI: 1.24–1.89, *Z* = 9.94, *P* < 0.00001) showed that knowledge scores improved significantly in BOPPPS teaching strategy with a large effect compared to LBL teaching. The random-effects model was used for the meta-analysis because of the significant statistical heterogeneity (*P* < 0.00001, *I*^2^ = 96% > 50%) among studies (Figure 5).

#### Measurements of comprehensive ability scores

**FIGURE 6:**
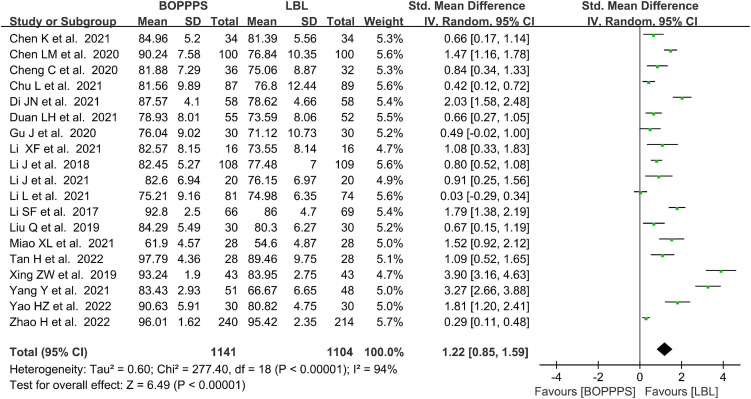
Forest plot of comprehensive ability scores for BOPPPS compared with LBL.

There were 19 included studies data related to the CAS evaluation with 1,141 and 1,104 students in the BOPPPS and LBL groups, respectively. In this meta-analysis, the CAS by a mean of 1.22 were significantly improved in the BOPPPS teaching strategy group compared with that of the traditional LBL model (95%CI: 0.85–1.59; *P* < 0.00001). The random-effects model was used for the meta-analysis because of the significant statistical heterogeneity (*P* < 0.00001, *I*^2^ = 94%) of the data (Figure 6).

#### Measurements of teaching satisfaction

**FIGURE 7:**
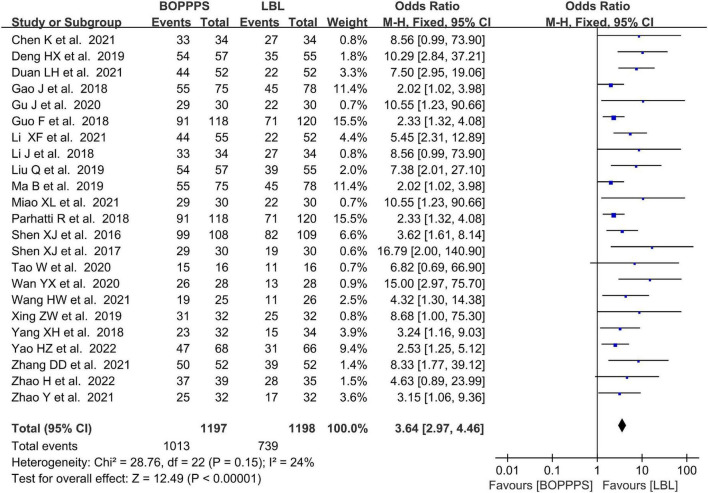
Forest plot of teaching satisfaction for BOPPPS compared with LBL.

There were 23 included studies data related to the satisfaction evaluation with 1,197 and 1,198 students in the BOPPPS and LBL groups, respectively. The meta-analysis in the BOPPPS group found a significantly higher odds ratio in TS compared with that of the LBL teaching (OR: 3.64; 95%CI: 2.97–4.46; *P* < 0.0001). The fixed-effects model was used for the meta-analysis because of the lower heterogeneity (*P* = 0.15, *I*^2^ = 24%) of the data (Figure 7).

### Subgroup analysis

**FIGURE 8:**
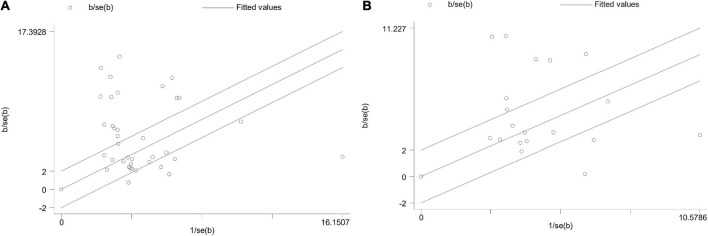
Galbraith plot analysis of heterogeneity. **(A)** Galbraith plot of knowledge examination scores; **(B)** Galbraith plot of comprehensive ability scores.

Galbraith plot of heterogeneity was shown in Figure 8 and the subgroup analysis was shown in Table 2 for KES and Table 3 for CAS (in Supplementary Appendixes 5, 6). Among subgroups aspects, we found differences regarding the efficiency of BOPPPS in the subgroups of attempted discipline contents and students training levels. The subgroup analysis demonstrated significant differences in the effects of BOPPPS on KES domain when comparing undergraduates and university graduates (*P* < 0.0001). The outcome revealed that there was a larger effect size (SMD 1.75, 95% CI 1.35–2.15) for undergraduates than that for university graduates (SMD 0.80, 95% CI 0.55–1.05). In addition, subgroup analysis of course contents showed that there were significant differences in the effects of BOPPPS on KES and CAS among 13 courses. The outcome demonstrated that the BOPPPS strategy was significantly superior to traditional LBL teaching in the courses teaching of pathology (SMD 3.13, 95% CI 0.77–5.50) and internal medicine (SMD 2.01, 95% CI 1.19–2.84).

**TABLE 2:** Subgroup analyses of final knowledge examination score in this meta-analysis.

Study characteristics	Participants			Test for heterogeneity			Test for effect		Subgroup
	Studies	BOPPPS	LBL	*I*^2^ (%)	Chi^2^ test	*P*-value	(SMD [CI])	*P*-value	Statistics, *P*-value
**1. Study design**									
RCT	30	1,634	1,608	94	451.89	<0.00001	1.54 [1.22, 1.87]	<0.00001	0.06, *P* = 0.81
Cohort study	7	934	873	98	287.71	<0.00001	1.65 [0.79, 2.52]	= 0.0002	
Total	37	2,568	2,481	96	900.97	<0.00001	1.56 [1.24, 1.89]	= 0.00001	
**2. Hometown**									
China students	34	2,449	2,391	96	868.60	<0.00001	1.56 [1.23, 1.90]	<0.00001	0.00, *P* = 0.98
Non-China students	3	119	90	92	26.38	<0.00001	1.58 [0.38, 2.78]	= 0.010	
Total	37	2,568	2,481	96	900.74	<0.00001	1.56 [0.38, 2.79]	<0.00001	
**3. Training levels**									
Undergraduates	30	2,123	2,072	97	879.76	<0.00001	1.75 [1.35, 2.15]	<0.00001	15.52, *P* < 0.0001
University graduates	7	430	409	55	13.28	= 0.04	0.80 [0.55, 1.05]	<0.00001	
Total	37	2,568	2,481	96	900.74	<0.00001	1.56 [1.24, 1.89]	<0.00001	
**4. Course type**									
Practice course	17	889	860	94	279.61	<0.00001	1.51 [1.04, 1.98]	<0.00001	0.08, *P* = 0.78
Theory course	20	1,679	1,621	97	589.74	<0.00001	1.61 [1.15, 2.06]	<0.00001	
Total	37	2,568	2,481	96	891.82	<0.00001	1.56 [1.24, 1.88]	<0.00001	
**5. Course contents**									
Anesthesiology	2	60	60	0	0.35	= 0.55	0.73 [0.36, 1.10]	= 0.0001	317.28, *P* < 0.00001
Diagnostics	5	217	204	93	55.74	<0.00001	1.25 [0.41, 2.10]	= 0.004	
Internal medicine	9	588	559	97	230.39	<0.00001	2.01 [1.19, 2.84]	<0.00001	
Obstetrics and gynecology	3	176	184	78	9.28	= 0.010	1.49 [0.91, 2.08]	<0.00001	
Surgery	6	320	314	87	39.27	<0.00001	1.05 [0.54, 1.55]	<0.0001	
Traditional Chinese medicine	4	219	155	78	13.51	= 0.0004	0.83 [0.35, 1.32]	= 0.0007	
Pathology	2	155	155	97	37.50	<0.00001	3.13 [0.77, 5.50]	= 0.009	
Emergency medicine	1	118	120				1.93[1.62, 2.23]	<0.00001	
Evidence-based medicine	1	25	25				2.84 [2.05, 3.64]	<0.00001	
General Medicine	1	57	55				5.80 [4.94, 6.66]	<0.00001	
Human anatomy	1	87	89				0.51 [0.21, 0.81]	= 0.0008	
Pediatrics	1	28	28				1.51 [0.91, 2.11]	<0.00001	
Physiology	1	518	523				0.22 [0.10, 0.34]	= 0.0003	
Total	37	2,568	2,481	96	836.18	<0.00001	1.57 [1.25, 1.88]	<0.00001	

**TABLE 3:** Subgroup analyses of comprehensive ability scores in this meta-analysis.

Study characteristics	Participants			Test for heterogeneity			Test for effect		Subgroup
	Studies	BOPPPS	LBL	*I*^2^ (%)	Chi^2^ test	*P*-value	(SMD [CI])	*P*-value	Statistics, *P*-value
**1. Training levels**									
Undergraduates	14	793	782	94	220.37	<0.00001	1.37 [0.91, 1.84]	<0.00001	3.64, *P* = 0.06
University graduates	5	348	322	78	18.39	= 0.001	0.75 [0.31, 1.19]	= 0.0008	
Total	19	1,141	1,104	94	277.40	<0.00001	1.22 [0.85, 1.59]	<0.00001	
**2. Course type**									
Practice course	14	767	741	92	166.37	<0.00001	1.25 [0.83, 1.67]	<0.00001	0.05, *P* = 0.82
Theory course	5	374	363	96	110.43	<0.00001	1.14 [0.30, 1.98]	= 0.0008	
Total	19	1,141	1,104	94	277.40	<0.00001	1.22 [0.85, 1.59]	<0.00001	
**3. Course contents**									
Diagnostics	3	133	122	74	7.55	= 0.02	0.63 [0.04, 1.22]	= 0.04	24.75, *P* = 0.002
Human anatomy	2	138	137	99	67.69	<0.00001	1.83 [−0.96, 4.63]	= 0.20	
Internal medicine	4	361	335	98	122.48	<0.00001	1.75 [0.27, 3.24]	= 0.02	
Pediatrics	2	94	97	74	3.90	= 0.05	1.47 [0.78, 2.15]	<0.0001	
Surgery	2	94	97	0	0.27	= 0.61	0.60 [0.29, 0.91]	= 0.0002	
Traditional Chinese medicine	3	94	94	80	10.17	= 0.006	1.02 [0.33, 1.72]	= 0.0002	
Anesthesiology	1	28	28				1.52 [0.92, 2.12]	<0.00001	
Obstetrics and gynecology	1	105	109				0.80 [0.52, 1.08]	<0.00001	
Pathology	1	100	100				1.47 [1.16, 1.78]	<0.00001	
Total	19	1,140	1,104	93	258.30	<0.00001	6.93 [4.43, 9.43]	<0.00001	

### Sensitivity analysis

A sensitivity analysis was performed to investigate the influence of each study on the overall meta-analysis summary estimate in Supplementary Appendix 7. Statistically similar results were obtained, which suggested the stability of this meta-analysis.

## Discussion

The greatest challenge for China medical schools is the training of qualified clinicians, which can both adapt to the hospital environment with rapidly changing and meet the needs of the Chinese people. Although the fact that the BOPPPS teaching strategy has been widely embraced in education systems around the world, its implementation in China is still in its infancy. Compared with lecture-based instruction, the BOPPPS teaching strategy is not a routine pedagogy in China medical education. Many factors such as medical education systems or cultural backgrounds maybe influence the selection of the BOPPPS teaching strategy. Firstly, the traditional teacher centered model has been dominating the Chinese medical education system. For both students and teachers, the traditional teaching model has almost formed prescribed educational experiences. Secondly, the lack of a high-quality and energetic teaching team is one of the important factors hindering the successful implementation of the BOPPPS teaching strategy. Thirdly, medical curriculum standards and education levels vary dramatically among different schools in China. Therefore, there is an urgent need to carry out a large number of educational reformations and rearrangements to successfully improve the Chinese medical education system.

We take into account the fact that the education systems, backgrounds of culture, history, and philosophy in China are distinct from those in western countries (58). To evaluate the potential effectiveness and differences in quantitative outcomes of the BOPPPS teaching strategy compared to the LBL teaching method in China, the target population in the current meta-analysis should be limited to medical students in Chinese medical schools.

In the study, 41 studies relevant to inclusion criteria were identified and demonstrated significant improvement with the BOPPPS teaching strategy in learning outcomes compared to traditional instructions. We analyzed the effectiveness of the BOPPPS teaching strategy in comparison with traditional lecture-based instruction using a meta-analysis to obtain a positive conclusion, which is the first meta-analysis providing comprehensive insights into the efficiency of the BOPPPS teaching strategy in Chinese medical education.

The meta-analysis results of the effectiveness indicator showed that when compared with the LBL group, the SS, KES, CAS, and TS of the BOPPPS group were higher, suggesting that the BOPPPS teaching strategy could stimulate the enthusiasm and interest of medical students, boost Students’ skills and intrinsic motivation in learning; and improve the self-directed learning ability, academic performance (6, 59). On the other hand, the BOPPPS teaching strategy with a six-phase framework was adapted to organize and accelerate the teaching cycle as a whole including goal, behavior, learning activity, and evaluation (60), which help teachers generate innovative teaching ideas and timely adjustment of teaching schedule. Besides, numerous knowledge points covered in medical education, the LBL methods do not encourage problem-solving and learning activities (61). The results of the meta-analysis showed that the students in the BOPPPS group demonstrated better mastery of the knowledge and greater active self-learning and comprehensive ability.

The pooled effect size of KES (SMD 1.56, 95% CI: 1.24–1.89), CAS (SMD 1.22, 95%CI: 0.85–1.59), SS (SMD 1.15, 95% CI: 1.00–1.30), and TS (OR: 3.64; 95%CI: 2.97–4.46) showed that BOPPPS learning remained more effective than traditional LBL learning. There are several possible explanations for this meta-analysis results. First, compared to traditional learning, the BOPPPS learning allowed students to review pre-assessment, post-assessment, and other materials as often as necessary, and this likely enhanced comprehensive ability and learning performance. Second, the BOPPPS teaching is a novelty model for Chinese medical students, and has greatly stimulated their interest and intrinsic motivation in subject learning. Students in the BOPPPS group appeared to be more proactive in course learning, which led them to obtain higher KES and TS. Third, although most Chinese medical schools use unified textbooks and syllabuses for all students, students in the BOPPPS group with definite learning objectives are more likely to get higher knowledge scores compared to the LBL group. Hence, the superiority of the BOPPPS teaching strategy relative to the traditional LBL teaching could be more evident according to comprehensive ability and knowledge performance.

For the outcome indicators of KES and CAS, there is a large heterogeneity among the included studies. The random-effects model was applied in that meta-analysis. Subgroup analyses consistently demonstrated that the KES and CAS were significantly different in students training levels and course contents. Although the effectiveness of the BOPPPS teaching strategy is very complex, those subgroup analyses partially explained the differences. This subgroup analysis revealed that the level of students training and the course contents may be much important factors affecting the effectiveness of the BOPPPS teaching. The knowledge level of students was dramatically different between undergraduates and university graduates. The curriculum teaching with the BOPPPS strategy should be based on the sound fundamental knowledge background and learning interests of students. Because university graduates have finished multidisciplinary medical courses, their focuses and interests are clinical practice and operational skills. For the undergraduates who completed part of the medicine course in schools, their learning enthusiasms and interests were easier to be stimulated with the BOPPPS teaching strategy (10), and thus improvement of academic performance.

Unlike LBL, the BOPPPS teaching strategy is not a routine pedagogy in medical education in many countries. This teaching strategy has a helpful six-phase framework for designing learning activities and thus can be used to assist teachers in disassembling and analyzing the teaching process to improve Students’ learning outcomes in medical education (62). In recent years, the BOPPPS teaching strategy has been extensively trialed in China’s higher education for the improvement of efficiency in medical education (6, 7, 10). Particularly, the COVID-19 pandemic has greatly impacted the education sector and the way of teaching and learning (63). Online teaching has become an important part of teaching strategy and thus, incorporation of the BOPPPS into a combined offline and online teaching should also be tried.

To better apply the BOPPPS teaching strategy in Chinese medical education environments, the important elements of the BOPPPS strategy should be considered before the class. First, a high-quality and qualified teaching team is needs for the successful implementation of BOPPPS teaching strategy. Second, teachers should select an appropriate curriculum content, and design a feasible teaching program based on teaching experiences that could improve the knowledge and clinical skill without excessive heavy course load and time for medical students. Third, the teaching strategy of BOPPPS is a two-dimensional cooperative relationship, which includes the dominant relationships between peers, students, and teachers. Students should be encouraged to participate in multimodal activities such as problem-gathering and discussion, self-assessment, and active feedback for the improvement of self-efficacy. Fourth, according to the BOPPPS teaching six-step, internet online-offline assisted teaching tools should be used to increase the communication opportunities with teachers and decrease the burden on teachers in the teaching activities. In addition, because of different learning obstacles and learning style preferences among undergraduate and postgraduate medical students, it is necessary to use teaching modalities targeting the specific characteristics of those Chinese medical students (64, 65). Before the class of BOPPPS teaching, students learning obstacles and style preferences should be evaluated. Correspondingly, teachers should remodel the BOPPPS teaching six-step, containing additional bridging course contexts, and classroom components of participatory learning for the improvement of teaching effectiveness.

However, there were also several limitations in the present systematic review and meta-analysis. First, the systematic literature search encompassed four English databases (PubMed, Web of Science, Embase, and Cochrane Library) and four Chinese databases (CNKI, Wanfang, CQVIP, and CBM) with inclusion and exclusion criteria. Second, the meta-analysis quality was dependent on the data quality from the included studies, and the included literature quality was with a low overall bias of 90.2% studies, and none of them described the allocation concealment or blinding sources. Third, there was a large heterogeneity among the included studies regarding KES and CAS. Results of subgroup analyses should be interpreted with caution because of the diversity of influencing factors. Fourth, there were no standard guidelines for the application of BOPPPS in medical disciplines and no standard criteria for the effective evaluation of BOPPPS teaching strategy in China. In addition, in some of the included studies, questionnaire surveys as an additional measurement were used to assess the comprehensive ability of BOPPPS in medical education. Finally, the participants included Chinese medical students and the study compared the effects of the BOPPPS teaching strategy and LBL only. It also needs to evaluate and compare the effectiveness of BOPPPS with other teaching methods with Bayesian network meta-analysis in the future.

In conclusion, the current meta-analysis demonstrates that the BOPPPS teaching strategy is more effective than traditional LBL methods in medical education for enhancing the knowledge scores, SS, CAS, as well as improving TS among Chinese medical students. The BOPPPS teaching strategy appears to be superior to LBL teaching, and the use of this model may be optimal for improving Chinese medical education. Based on the limitations of this meta-analysis, we believe that high-quality studies with well-designed RCTs to assess the effectiveness of the BOPPPS teaching strategy are needed in the Chinese medical education system in the future.

## Data availability statement

The original contributions presented in this study are included in the article/Supplementary material, further inquiries can be directed to the corresponding author.

## Author contributions

XM performed the design and conceived the original idea. XM and DZ were responsible for the drafting of the manuscript. JW and KX were responsible for the literature search, data extraction, and statistical analysis. LL supervised the study and coordinated the teaching management. All authors have read and approved the final manuscript.
