# Supplementary material for: Effectiveness of bridge-in, objective, pre-assessment, participatory learning, post-assessment, and summary teaching strategy in Chinese medical education: A systematic review and meta-analysis
Source: Front Med (Lausanne). 2022 Sep 15;9:975229. doi: 10.3389/fmed.2022.975229 (PMC9521335; doi:10.3389/fmed.2022.975229)

Supplementary material

**Appendix 1**	The flowchart of class design for the BOPPPS model
**Appendix 2**	PRISMA checklist
**Appendix 3**	PubMed and CNKI literature search strategy
**Appendix 4**	The quality assessment of each RCT with RoB2 tool
**Appendix 5**	Forest plot of subgroup analyses of final knowledge examination score
**Appendix 6**	Forest plot of subgroup analyses of comprehensive ability score
**Appendix 7**	Sensitive analysis of all the studies using Metaninf function in Stata

Appendix 1. The flow chart of class design for the BOPPPS model

**Phase**	**Teaching Content**
Bridge-in	A diverse way of class introduction is used to attract the students' attention, to generate strong motivation, and to generate interest of further learning content.
Objective	The learning objectives of each class have the guiding and evaluation functions, so that the students can clearly understand the content of a lesson and their learning outcomes at the end of the course.
Pre-assessment	The pre-assessment phase reviews the concepts that students already know about the course and determines the starting point of learning. According to their performances in the preassessment, the depth and progress of the learning content will improve over time.
Participatory learning	Participatory learning is the main body of learning activities. It emphasizes on full interaction between students and classmates and/or teachers, which guides an active learning atmosphere.
Post-assessment	The students' learning effect is evaluated in this phase. Through feedback from the learning activities, we can understand the extent to which those students achieved the learning objective.
Summary	For teachers and students, summaries provide an opportunity to reflect on what they have learned in class.

For the traditional lecture-based learning, the students firstly received the reading material including textbooks and syllabus of course. Then, the overhead projector was used to teach in the face-to-face classroom with the student passive receiving the associated knowledge in each class. Students had opportunities to use teaching materials and reference books which were also used by students of the BOPPPS model. Finally, the teachers answered students' questions and repeated any knowledge points that they had not been fully understood in the class.

Appendix 2. PRISMA checklist

Section/topic	Item No	Checklist item	Reported on page No
**Title**			
Title	1	Identify the report as a systematic review, meta-analysis, or both	1
**Abstract**			
Structured summary	2	Provide a structured summary including, as applicable, background, objectives, data sources, study eligibility criteria, participants, interventions, study appraisal and synthesis methods, results, limitations, conclusions and implications of key findings, systematic review registration number	2
**Introduction**			
Rationale	3	Describe the rationale for the review in the context of what is already known	Introduction
Objectives	4	Provide an explicit statement of questions being addressed with reference to participants, interventions, comparisons, outcomes, and study design (PICOS)	Introduction
**Methods**			
Protocol and registration	5	Indicate if a review protocol exists, if and where it can be accessed (such as web address), and, if available, provide registration information including registration number	No
Eligibility criteria	6	Specify study characteristics (such as PICOS, length of follow-up) and report characteristics (such as years considered, language, publication status) used as criteria for eligibility, giving rationale	Methods
Information sources	7	Describe all information sources (such as databases with dates of coverage, contact with study authors to identify additional studies) in the search and date last searched	Methods
Search	8	Present full electronic search strategy for at least one database, including any limits used, such that it could be repeated	Appendix 3
Study selection	9	State the process for selecting studies (that is, screening, eligibility, included in systematic review, and, if applicable, included in the meta-analysis)	Methods
Data collection process	10	Describe method of data extraction from reports (such as piloted forms, independently, in duplicate) and any processes for obtaining and confirming data from investigators	Methods
Data items	11	List and define all variables for which data were sought (such as PICOS, funding sources) and any assumptions and simplifications made	Methods
Risk of bias in individual studies	12	Describe methods used for assessing risk of bias of individual studies (including specification of whether this was done at the study or outcome level), and how this information is to be used in any data synthesis	Methods
Summary measures	13	State the principal summary measures (such as risk ratio, difference in means).	Methods
Synthesis of results	14	Describe the methods of handling data and combining results of studies, if done, including measures of consistency (such as I^2 statistic) for each meta-analysis	Methods
Risk of bias across studies	15	Specify any assessment of risk of bias that may affect the cumulative evidence (such as publication bias, selective reporting within studies)	Methods
Additional analyses	16	Describe methods of additional analyses (such as sensitivity or subgroup analyses, meta-regression), if done, indicating which were pre-specified	Methods
**Results**			
Study selection	17	Give numbers of studies screened, assessed for eligibility, and included in the review, with reasons for exclusions at each stage, ideally with a flow diagram	Figure 1

Section/topic	Item No	Checklist item	Reported on page No
Study characteristics	18	For each study, present characteristics for which data were extracted (such as study size, PICOS, follow-up period) and provide the citations	Table 1
Risk of bias within studies	19	Present data on risk of bias of each study and, if available, any outcome-level assessment (see item 12).	Figure 2, Appendix 4
Results of individual studies	20	For all outcomes considered (benefits or harms), present for each study (a) simple summary data for each intervention group and (b) effect estimates and confidence intervals, ideally with a forest plot	Figure 4-7
Synthesis of results	21	Present results of each meta-analysis done, including confidence intervals and measures of consistency	Figures 4-7
Risk of bias across studies	22	Present results of any assessment of risk of bias across studies (see item 15)	Figure 3
Additional analysis	23	Give results of additional analyses, if done (such as sensitivity or subgroup analyses, meta-regression) (see item 16)	Table 2-3, Figures 8, Appendix 5-7
**Discussion**			
Summary of evidence	24	Summarise the main findings including the strength of evidence for each main outcome; consider their relevance to key groups (such as health care providers, users, and policy makers)	Discussion
Limitations	25	Discuss limitations at study and outcome level (such as risk of bias), and at review level (such as incomplete retrieval of identified research, reporting bias)	Discussion
Conclusions	26	Provide a general interpretation of the results in the context of other evidence, and implications for future research	Discussion
**Funding**			
Funding	27	Describe sources of funding for the systematic review and other support (such as supply of data) and role of funders for the systematic review	Page 26

Appendix 3. The research strategy of PubMed and CNKI

Pubmed search strategy (before 6 Jun 2022)

#5	#3 AND #4	5
#4	#1 OR #2	5
#3	student*	422,159
#2	(((((bridge[Title/Abstract]) AND (objective[Title/Abstract])) AND (preassessment[Title/Abstract])) AND (participatory[Title/Abstract])) AND (postassessment[Title/Abstract])) AND (summary[Title/Abstract])	1
#1	BOPPPS	5

CNKI search strategy (before 6 Jun 2022)

#4 #1 AND #2 AND #3 58

#3 (Subject=Control) OR (Title/Keyword/Abstract =Control)

#2 (Subject=Medical) OR (Title/Keyword/Abstract =Medical)

#1 (Subject=BOPPPS) OR (Title/Keyword/Abstract =BOPPPS)

Summary (before 6 Jun 2022)

Web of Science=26

PubMed=5

Embase=7

Cochrane Library=2

CNKI=58

CQVIP=97

Wanfang=97

CBM=75

Appendix 4. The quality assessment of each RCT (n=41) with RoB2 tool

	Randomization process	Deviations from intended interventions	Mising outcome data	Measurement of the outcome	Selection of the reported result	Overall Bias
Low risk	97.6	100	97.6	100	95.1	90.2
Some concerns	2.4	0	2.4	0	4.9	9.8
High risk	0	0	0	0	0	0

Unique ID	Study ID	Weight	D1	D2	D3	D4	D5	Overall
m1	Chen K et al. 2021	1	+	+	+	+	+	+
m2	Chen LM et al. 2020	1	+	+	+	+	+	+
m3	Cheng C et al. 2020	1	+	+	+	+	+	+
m4	Chu L et al. 2021	1	+	+	+	+	+	+
m5	Deng HX et al. 2019	1	+	+	+	+	+	+
m6	Di JN et al. 2021	1	+	+	!	+	+	!
m7	Duan LH et al. 2021	1	+	+	+	+	+	+
m8	Gao J et al. 2018	1	+	+	+	+	+	+
m9	Gu J et al. 2020	1	+	+	+	+	+	+
m10	Guo F et al. 2018	1	+	+	+	+	+	+
m11	Li XF et al. 2021	1	+	+	+	+	+	+
m12	Li J et al. 2018	1	+	+	+	+	+	+
m13	Li J et al. 2021	1	+	+	+	+	!	!
m14	Li L et al. 2021	1	+	+	+	+	+	+
m15	Li S et al. 2022	1	+	+	+	+	+	+
m16	Li SF et al. 2017	1	+	+	+	+	!	!
m17	Liu Q et al. 2019	1	+	+	+	+	+	+
m18	Liu XY et al 2022	1	!	+	+	+	+	!
m19	Liu YD et al. 2020	1	+	+	+	+	+	+
m20	Ma B et al. 2019	1	+	+	+	+	+	+
m21	Miao XL et al. 2021	1	+	+	+	+	+	+
m22	Parhatti R et al. 20	1	+	+	+	+	+	+
m23	Qin YH et al. 2021	1	+	+	+	+	+	+
m24	Shen XJ et al. 2016	1	+	+	+	+	+	+
m25	Shen XJ et al. 2017	1	+	+	+	+	+	+
m26	Sun YR et al. 2020	1	+	+	+	+	+	+
m27	Tan H et al. 2022	1	+	+	+	+	+	+
m28	Tao W et al. 2020	1	+	+	+	+	+	+
m29	Wan YX et al. 2020	1	+	+	+	+	+	+
m30	Wang HW et al. 20	1	+	+	+	+	+	+
m31	Xing ZW et al. 2019	1	+	+	+	+	+	+
m32	Xu YJ et al. 2019	1	+	+	+	+	+	+
m33	Yang XH et al. 2018	1	+	+	+	+	+	+
m34	Yang Y et al. 2021	1	+	+	+	+	+	+
m35	Yao HZ et al. 2022	1	+	+	+	+	+	+
m36	Zhang DD et al. 202	1	+	+	+	+	+	+
m37	Zhang YY et al. 201	1	+	+	+	+	+	+
m38	Zhao H et al. 2022	1	+	+	+	+	+	+
m39	Zhao Y et al. 2021	1	+	+	+	+	+	+
m40	Zhou Y et al. 2020	1	+	+	+	+	+	+
m41	Zhou Y et al. 2021	1	+	+	+	+	+	+

+ Low risk
! Some concerns
\- High risk

D1 Randomisation process
D2 Deviations from the intended interventions
D3 Missing outcome data
D4 Measurement of the outcome
D5 Selection of the reported result

Appendix 5. Forest plot of subgroup analyses of final knowledge examination score

5.1 Subgroup analyses of study design

Study or Subgroup	BOPPPS Mean	BOPPPS SD	BOPPPS Total	LBL Mean	LBL SD	LBL Total	Weight	Std. Mean Difference IV, Random, 95% CI
**1.9.1 RCT**								
Chen K et al. 2021	85.91	4.58	34	83.03	5.72	34	2.7%	0.55 [0.06, 1.03]
Chen LM et al. 2020	89.79	10.52	100	72.94	6.18	100	2.8%	1.95 [1.61, 2.28]
Cheng C et al. 2020	80.37	8.97	36	73.81	9.97	32	2.7%	0.69 [0.20, 1.18]
Chu L et al. 2021	83.95	7.42	87	79.37	10.16	89	2.8%	0.51 [0.21, 0.81]
Di JN et al. 2021	83.62	4.64	58	80.53	4.52	58	2.8%	0.67 [0.30, 1.04]
Duan LH et al. 2021	76.56	8.54	55	71.69	7.82	52	2.8%	0.59 [0.20, 0.98]
Gao J et al. 2018	87.61	1.36	75	81.03	1.64	78	2.7%	4.34 [3.75, 4.92]
Gu J et al. 2020	76.4	10.34	30	74.4	10.2	30	2.7%	0.19 [-0.32, 0.70]
Guo F et al. 2018	88.78	5.65	118	79.12	4.27	120	2.8%	1.93 [1.62, 2.23]
Li XF et al. 2021	85.65	8.84	16	72.75	8.25	16	2.5%	1.47 [0.68, 2.26]
Li J et al. 2021	86.05	5.84	20	79.3	6.42	20	2.6%	1.08 [0.41, 1.75]
Liu YD et al. 2020	81.33	9.73	36	73.21	9.98	36	2.7%	0.82 [0.33, 1.30]
Ma B et al. 2019	88.1	4.7	25	63.3	11.1	26	2.5%	2.84 [2.05, 3.64]
Miao XL et al. 2021	85.38	9.45	28	76.9	10.2	28	2.7%	0.85 [0.30, 1.40]
Parhatti R et al. 2018	89.83	5.6	32	78.65	5.49	32	2.6%	1.99 [1.39, 2.60]
Qin YH et al. 2021	86.2	5.12	15	81.7	5.73	15	2.5%	0.81 [0.06, 1.55]
Shen XJ et al. 2016	79.91	8.89	32	74.5	9.54	34	2.7%	0.58 [0.09, 1.07]
Tan H et al. 2022	79.32	6.2	28	66.18	10.43	28	2.7%	1.51 [0.91, 2.11]
Tao W et al. 2020	86.53	5.98	52	79.67	5.43	52	2.8%	1.19 [0.77, 1.61]
Wan YX et al. 2020	87.69	11.57	32	78.42	17.21	32	2.7%	0.62 [0.12, 1.13]
Wang HW et al. 2021	94.32	2.11	39	84.39	2.28	35	2.4%	4.48 [3.61, 5.35]
Xing ZW et al. 2019	92.05	2.23	43	82.25	3.21	43	2.6%	3.51 [2.83, 4.20]
Yang XH et al. 2018	89.23	5.76	116	78.93	7.89	119	2.9%	1.48 [1.19, 1.77]
Yao HZ et al. 2022	91.03	5.96	30	81.22	4.82	30	2.6%	1.79 [1.18, 2.39]
Zhang DD et al. 2021	90.46	5.31	30	74.98	8.36	30	2.6%	2.18 [1.53, 2.83]
Zhang YY et al. 2018	80.97	7.21	30	74.27	7.4	35	2.7%	0.91 [0.39, 1.42]
Zhao H et al. 2022	89.44	8.41	240	82.76	10.08	214	2.9%	0.72 [0.53, 0.91]
Zhao Y et al. 2021	83.61	8.16	115	70.35	9.32	112	2.8%	1.51 [1.21, 1.81]
Zhou Y et al. 2020	88.27	2.73	30	71.23	9.87	30	2.6%	2.32 [1.66, 2.99]
Zhou Y et al. 2021	72.28	1.93	52	64.62	2.71	48	2.6%	3.25 [2.65, 3.86]
**Subtotal (95% CI)**			**1634**			**1608**	**80.8%**	**1.54 [1.22, 1.87]**
Heterogeneity: Tau² = 0.73; Chi² = 451.89, df = 29 (P < 0.00001); I² = 94%								
Test for overall effect: Z = 9.44 (P < 0.00001)								
**1.9.2 Cohort study**								
Deng HX et al. 2019	81.39	1.65	57	71.31	1.8	55	2.4%	5.80 [4.94, 6.66]
Li L et al. 2021	73.92	6.99	81	71.86	8.44	74	2.8%	0.27 [-0.05, 0.58]
Li S et al. 2022	75.79	10.15	57	69.64	16.79	28	2.8%	0.48 [0.02, 0.94]
Liu XY et al 2022	83.9	11.38	518	81.1	13.72	532	2.9%	0.22 [0.10, 0.34]
Shen XJ et al. 2017	79.99	8.31	68	76.11	9.68	66	2.8%	0.43 [0.09, 0.77]
Sun YR et al. 2020	46.03	2.31	55	34.85	2.76	55	2.6%	4.36 [3.67, 5.06]
Xu YJ et al. 2019	76.1	13.19	98	66.02	17.35	63	2.8%	0.67 [0.35, 1.00]
**Subtotal (95% CI)**			**934**			**873**	**19.2%**	**1.65 [0.79, 2.52]**
Heterogeneity: Tau² = 1.29; Chi² = 287.71, df = 6 (P < 0.00001); I² = 98%								
Test for overall effect: Z = 3.77 (P = 0.0002)								
**Total (95% CI)**			**2568**			**2481**	**100.0%**	**1.56 [1.24, 1.89]**
Heterogeneity: Tau² = 0.93; Chi² = 900.74, df = 36 (P < 0.00001); I² = 96%								
Test for overall effect: Z = 9.48 (P < 0.00001)								
Test for subgroup differences: Chi² = 0.06, df = 1 (P = 0.81), I² = 0%								

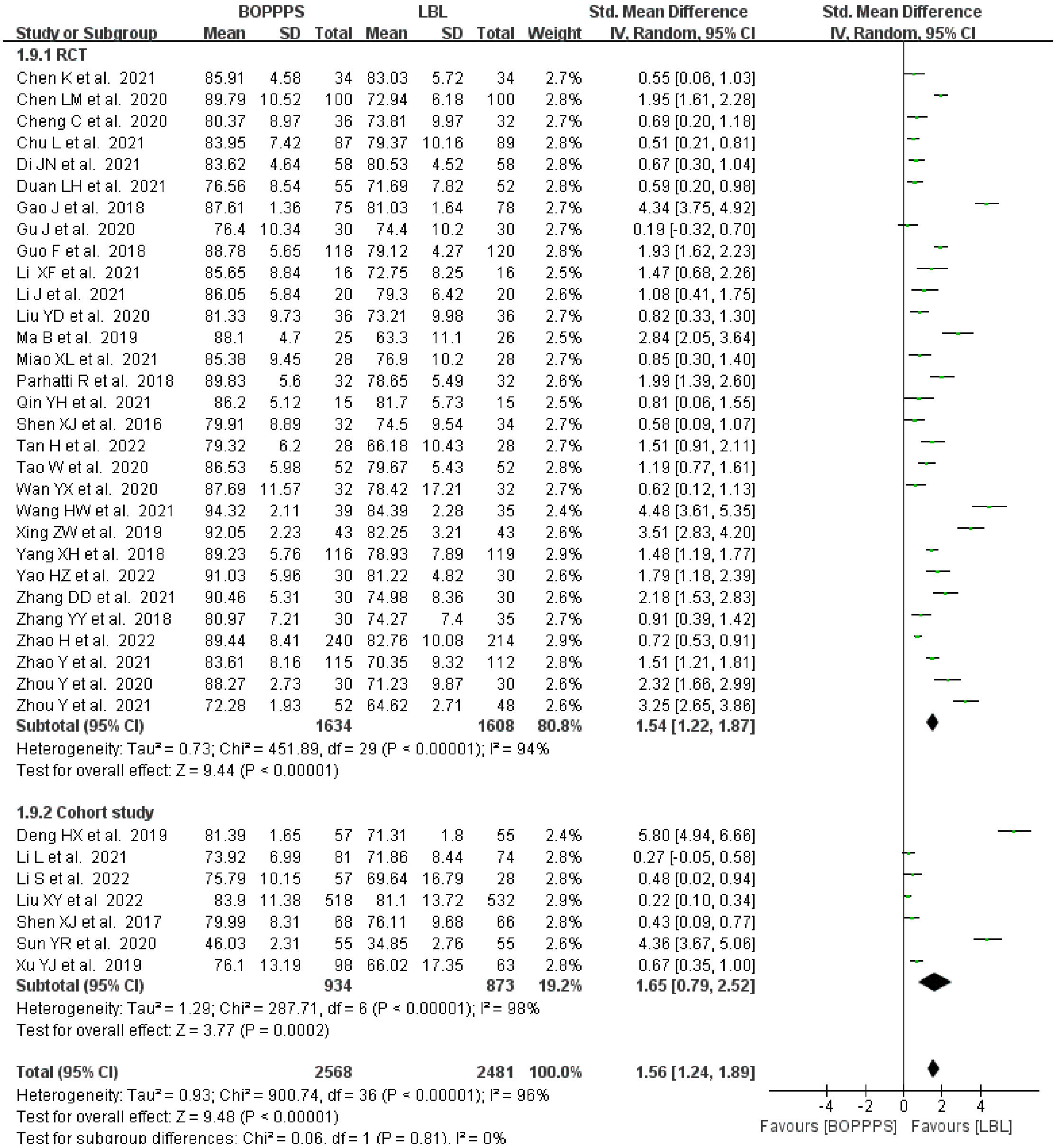

Appendix 5. Forest plot of subgroup analyses of final knowledge examination score

5.2 Subgroup analyses of hometown

Study or Subgroup	BOPPPS Mean	BOPPPS SD	BOPPPS Total	LBL Mean	LBL SD	LBL Total	Weight	Std. Mean Difference IV, Random, 95% CI
**1.5.1 China students**								
Chen K et al. 2021	80.37	8.97	36	73.81	9.97	32	2.7%	0.69 [0.20, 1.18]
Chen LM et al. 2020	85.91	4.58	34	83.03	5.72	34	2.7%	0.55 [0.06, 1.03]
Cheng C et al. 2020	89.79	10.52	100	72.94	6.18	100	2.8%	1.95 [1.61, 2.28]
Chu L et al. 2021	83.95	7.42	87	79.37	10.16	89	2.8%	0.51 [0.21, 0.81]
Deng HX et al. 2019	81.39	1.65	57	71.31	1.8	55	2.4%	5.80 [4.94, 6.66]
Di JN et al. 2021	83.62	4.64	58	80.53	4.52	58	2.8%	0.67 [0.30, 1.04]
Duan LH et al. 2021	76.56	8.54	55	71.69	7.82	52	2.8%	0.59 [0.20, 0.98]
Gao J et al. 2018	87.61	1.36	75	81.03	1.64	78	2.7%	4.34 [3.75, 4.92]
Gu J et al. 2020	76.4	10.34	30	74.4	10.2	30	2.7%	0.19 [-0.32, 0.70]
Guo F et al. 2018	88.78	5.65	118	79.12	4.27	120	2.8%	1.93 [1.62, 2.23]
Li XF et al. 2021	86.05	5.84	20	79.3	6.42	20	2.6%	1.08 [0.41, 1.75]
Li J et al. 2018	73.92	6.99	81	71.86	8.44	74	2.8%	0.27 [-0.05, 0.58]
Li L et al. 2021	83.9	11.38	518	81.1	13.72	532	2.9%	0.22 [0.10, 0.34]
Liu XY et al 2022	81.33	9.73	36	73.21	9.98	36	2.7%	0.82 [0.33, 1.30]
Liu YD et al. 2020	85.65	8.84	16	72.75	8.25	16	2.5%	1.47 [0.68, 2.26]
Ma B et al. 2019	88.1	4.7	25	63.3	11.1	26	2.5%	2.84 [2.05, 3.64]
Miao XL et al. 2021	85.38	9.45	28	76.9	10.2	28	2.7%	0.85 [0.30, 1.40]
Qin YH et al. 2021	86.2	5.12	15	81.7	5.73	15	2.5%	0.81 [0.06, 1.55]
Shen XJ et al. 2016	79.91	8.89	32	74.5	9.54	34	2.7%	0.58 [0.09, 1.07]
Shen XJ et al. 2017	79.99	8.31	68	76.11	9.68	66	2.8%	0.43 [0.09, 0.77]
Sun YR et al. 2020	46.03	2.31	55	34.85	2.76	55	2.6%	4.36 [3.67, 5.06]
Tan H et al. 2022	79.32	6.2	28	66.18	10.43	28	2.7%	1.51 [0.91, 2.11]
Tao W et al. 2020	86.53	5.98	52	79.67	5.43	52	2.8%	1.19 [0.77, 1.61]
Wan YX et al. 2020	94.32	2.11	39	84.39	2.28	35	2.4%	4.48 [3.61, 5.35]
Wang HW et al. 2021	87.69	11.57	32	78.42	17.21	32	2.7%	0.62 [0.12, 1.13]
Xing ZW et al. 2019	92.05	2.23	43	82.25	3.21	43	2.6%	3.51 [2.83, 4.20]
Xu YJ et al. 2019	76.1	13.19	98	66.02	17.35	63	2.8%	0.67 [0.35, 1.00]
Yang XH et al. 2018	89.23	5.76	116	78.93	7.89	119	2.9%	1.48 [1.19, 1.77]
Yao HZ et al. 2022	91.03	5.96	30	81.22	4.82	30	2.6%	1.79 [1.18, 2.39]
Zhang DD et al. 2021	90.46	5.31	30	74.98	8.36	30	2.6%	2.18 [1.53, 2.83]
Zhang YY et al. 2018	80.97	7.21	30	74.27	7.4	35	2.7%	0.91 [0.39, 1.42]
Zhao H et al. 2022	89.44	8.41	240	82.76	10.08	214	2.9%	0.72 [0.53, 0.91]
Zhao Y et al. 2021	83.61	8.16	115	70.35	9.32	112	2.8%	1.51 [1.21, 1.81]
Zhou Y et al. 2020	72.28	1.93	52	64.62	2.71	48	2.6%	3.25 [2.65, 3.86]
**Subtotal (95% CI)**			**2449**			**2391**	**92.0%**	**1.56 [1.23, 1.90]**
Heterogeneity: Tau² = 0.94; Chi² = 868.60, df = 33 (P < 0.00001); I² = 96%								
Test for overall effect: Z = 9.05 (P < 0.00001)								
**1.5.2 Non-China students**								
Li S et al. 2022	75.79	10.15	57	69.64	16.79	28	2.8%	0.48 [0.02, 0.94]
Parhatti R et al. 2018	89.83	5.6	32	78.65	5.49	32	2.6%	1.99 [1.39, 2.60]
Zhou Y et al. 2021	88.27	2.73	30	71.23	9.87	30	2.6%	2.32 [1.66, 2.99]
**Subtotal (95% CI)**			**119**			**90**	**8.0%**	**1.58 [0.38, 2.78]**
Heterogeneity: Tau² = 1.03; Chi² = 26.38, df = 2 (P < 0.00001); I² = 92%								
Test for overall effect: Z = 2.58 (P = 0.010)								
**Total (95% CI)**			**2568**			**2481**	**100.0%**	**1.56 [1.24, 1.89]**
Heterogeneity: Tau² = 0.93; Chi² = 900.74, df = 36 (P < 0.00001); I² = 96%								
Test for overall effect: Z = 9.48 (P < 0.00001)								
Test for subgroup differences: Chi² = 0.00, df = 1 (P = 0.98), I² = 0%								

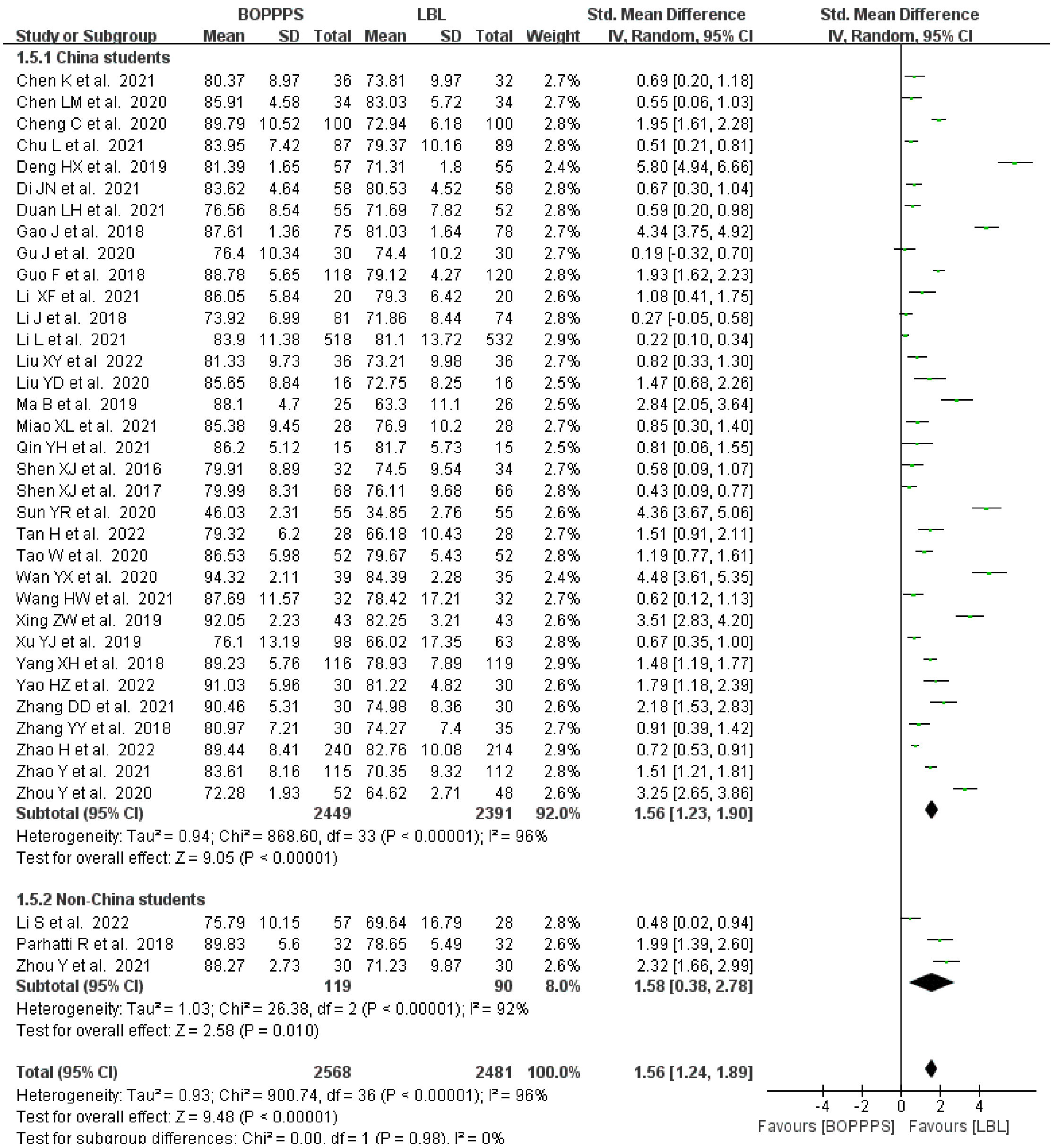

Appendix 5. Forest plot of subgroup analyses of final knowledge examination score

5.3 Subgroup analyses of training levels

Study or Subgroup	BOPPPS Mean	BOPPPS SD	BOPPPS Total	LBL Mean	LBL SD	LBL Total	Weight	Std. Mean Difference IV, Random, 95% CI
**1.6.1 Undergraduates**								
Chen LM et al. 2020	89.79	10.52	100	72.94	6.18	100	2.8%	1.95 [1.61, 2.28]
Cheng C et al. 2020	80.37	8.97	36	73.81	9.97	32	2.7%	0.69 [0.20, 1.18]
Chu L et al. 2021	83.95	7.42	87	79.37	10.16	89	2.8%	0.51 [0.21, 0.81]
Deng HX et al. 2019	81.39	1.65	57	71.31	1.8	55	2.4%	5.80 [4.94, 6.66]
Di JN et al. 2021	83.62	4.64	58	80.53	4.52	58	2.8%	0.67 [0.30, 1.04]
Duan LH et al. 2021	76.56	8.54	55	71.69	7.82	52	2.8%	0.59 [0.20, 0.98]
Gao J et al. 2018	87.61	1.36	75	81.03	1.64	78	2.7%	4.34 [3.75, 4.92]
Guo F et al. 2018	88.78	5.65	118	79.12	4.27	120	2.8%	1.93 [1.62, 2.23]
Li J et al. 2021	86.05	5.84	20	79.3	6.42	20	2.6%	1.08 [0.41, 1.75]
Li L et al. 2021	73.92	6.99	81	71.86	8.44	74	2.8%	0.27 [-0.05, 0.58]
Li S et al. 2022	75.79	10.15	57	69.64	16.79	28	2.8%	0.48 [0.02, 0.94]
Liu XY et al 2022	83.9	11.38	518	81.1	13.72	532	2.9%	0.22 [0.10, 0.34]
Liu YD et al. 2020	81.33	9.73	36	73.21	9.98	36	2.7%	0.82 [0.33, 1.30]
Ma B et al. 2019	88.1	4.7	25	63.3	11.1	26	2.5%	2.84 [2.05, 3.64]
Parhatti R et al. 2018	89.83	5.6	32	78.65	5.49	32	2.6%	1.99 [1.39, 2.60]
Qin YH et al. 2021	86.2	5.12	15	81.7	5.73	15	2.5%	0.81 [0.06, 1.55]
Shen XJ et al. 2016	79.91	8.89	32	74.5	9.54	34	2.7%	0.58 [0.09, 1.07]
Shen XJ et al. 2017	79.99	8.31	68	76.11	9.68	66	2.8%	0.43 [0.09, 0.77]
Sun YR et al. 2020	46.03	2.31	55	34.85	2.76	55	2.6%	4.36 [3.67, 5.06]
Tan H et al. 2022	79.32	6.2	28	66.18	10.43	28	2.7%	1.51 [0.91, 2.11]
Wan YX et al. 2020	87.69	11.57	32	78.42	17.21	32	2.7%	0.62 [0.12, 1.13]
Wang HW et al. 2021	94.32	2.11	39	84.39	2.28	35	2.4%	4.48 [3.61, 5.35]
Xing ZW et al. 2019	92.05	2.23	43	82.25	3.21	43	2.6%	3.51 [2.83, 4.20]
Xu YJ et al. 2019	76.1	13.19	98	66.02	17.35	63	2.8%	0.67 [0.35, 1.00]
Yang XH et al. 2018	89.23	5.76	116	78.93	7.89	119	2.9%	1.48 [1.19, 1.77]
Yao HZ et al. 2022	91.03	5.96	30	81.22	4.82	30	2.6%	1.79 [1.18, 2.39]
Zhang DD et al. 2021	90.46	5.31	30	74.98	8.36	30	2.6%	2.18 [1.53, 2.83]
Zhao Y et al. 2021	83.61	8.16	115	70.35	9.32	112	2.8%	1.51 [1.21, 1.81]
Zhou Y et al. 2020	88.27	2.73	30	71.23	9.87	30	2.6%	2.32 [1.66, 2.99]
Zhou Y et al. 2021	72.28	1.93	52	64.62	2.71	48	2.6%	3.25 [2.65, 3.86]
**Subtotal (95% CI)**			**2138**			**2072**	**81.0%**	**1.75 [1.35, 2.15]**
Heterogeneity: $Tau^2 = 1.17$; $Chi^2 = 879.76$, df = 29 ($P < 0.00001$); $I^2 = 97\%$								
Test for overall effect: Z = 8.57 ($P < 0.00001$)								
**1.6.2 Graduates**								
Chen K et al. 2021	85.91	4.58	34	83.03	5.72	34	2.7%	0.55 [0.06, 1.03]
Gu J et al. 2020	76.4	10.34	30	74.4	10.2	30	2.7%	0.19 [-0.32, 0.70]
Li XF et al. 2021	85.65	8.84	16	72.75	8.25	16	2.5%	1.47 [0.68, 2.26]
Miao XL et al. 2021	85.38	9.45	28	76.9	10.2	28	2.7%	0.85 [0.30, 1.40]
Tao W et al. 2020	86.53	5.98	52	79.67	5.43	52	2.8%	1.19 [0.77, 1.61]
Zhang YY et al. 2018	80.97	7.21	30	74.27	7.4	35	2.7%	0.91 [0.39, 1.42]
Zhao H et al. 2022	89.44	8.41	240	82.76	10.08	214	2.9%	0.72 [0.53, 0.91]
**Subtotal (95% CI)**			**430**			**409**	**19.0%**	**0.80 [0.55, 1.05]**
Heterogeneity: $Tau^2 = 0.06$; $Chi^2 = 13.28$, df = 6 ($P = 0.04$); $I^2 = 55\%$								
Test for overall effect: Z = 6.24 ($P < 0.00001$)								
**Total (95% CI)**			**2568**			**2481**	**100.0%**	**1.56 [1.24, 1.89]**
Heterogeneity: $Tau^2 = 0.93$; $Chi^2 = 900.74$, df = 36 ($P < 0.00001$); $I^2 = 96\%$								
Test for overall effect: Z = 9.48 ($P < 0.00001$)								
Test for subgroup differences: $Chi^2 = 15.54$, df = 1 ($P < 0.0001$), $I^2 = 93.6\%$								

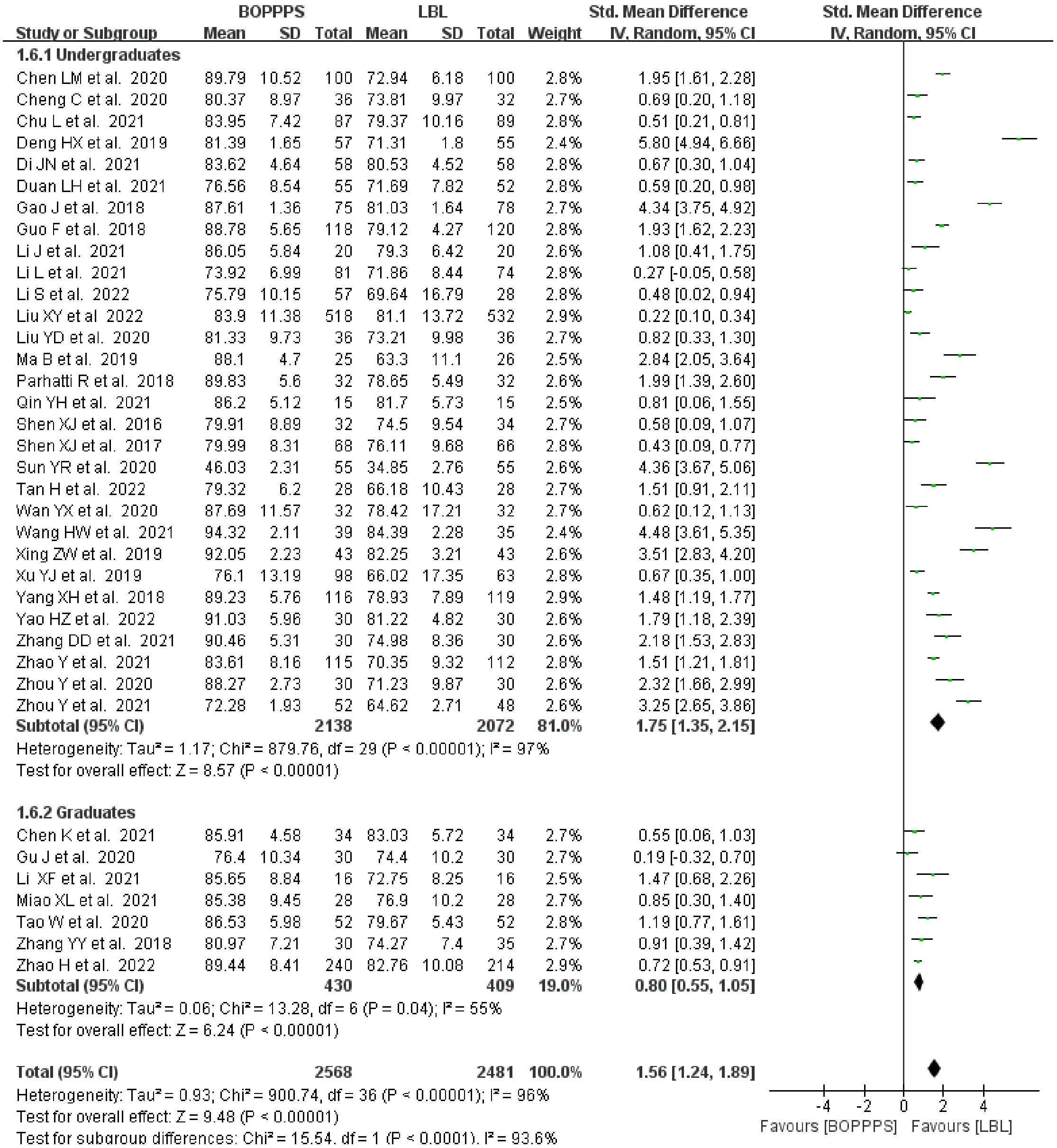

Appendix 5. Forest plot of subgroup analyses of final knowledge examination score

5.4 Subgroup analyses of course type

	BOPPPS			LBL				Std. Mean Difference
Study or Subgroup	Mean	SD	Total	Mean	SD	Total	Weight	IV, Random, 95% CI
**1.7.1 Practice course**								
Chen K et al. 2021	85.91	4.58	34	83.03	5.72	34	2.7%	0.55 [0.06, 1.03]
Cheng C et al. 2020	80.37	8.97	36	73.81	9.97	32	2.7%	0.69 [0.20, 1.18]
Di JN et al. 2021	83.62	4.64	58	80.53	4.52	58	2.8%	0.67 [0.30, 1.04]
Gao J et al. 2018	87.61	1.36	75	81.03	1.64	78	2.7%	4.34 [3.75, 4.92]
Gu J et al. 2020	76.4	10.34	30	74.4	10.2	30	2.7%	0.19 [-0.32, 0.70]
Li XF et al. 2021	85.65	8.84	16	72.75	8.25	16	2.5%	1.47 [0.68, 2.26]
Li J et al. 2021	86.05	5.84	20	79.3	6.42	20	2.6%	1.08 [0.41, 1.75]
Miao XL et al. 2021	85.38	9.45	28	76.9	10.2	28	2.7%	0.85 [0.30, 1.40]
Qin YH et al. 2021	86.2	5.12	15	81.7	5.73	15	2.5%	0.81 [0.06, 1.55]
Tan H et al. 2022	79.32	6.2	28	66.18	10.43	28	2.7%	1.51 [0.91, 2.11]
Tao W et al. 2020	86.53	5.98	52	79.67	5.43	52	2.8%	1.19 [0.77, 1.61]
Wang HW et al. 2021	94.32	2.11	39	84.39	2.28	35	2.4%	4.48 [3.61, 5.35]
Xing ZW et al. 2019	92.05	2.23	43	82.25	3.21	43	2.6%	3.51 [2.83, 4.20]
Yao HZ et al. 2022	91.03	5.96	30	81.22	4.82	30	2.6%	1.79 [1.18, 2.39]
Zhang YY et al. 2018	80.97	7.21	30	74.27	7.4	35	2.7%	0.91 [0.39, 1.42]
Zhao H et al. 2022	89.44	8.41	240	82.76	10.08	214	2.9%	0.72 [0.53, 0.91]
Zhao Y et al. 2021	83.61	8.16	115	70.35	9.32	112	2.9%	1.51 [1.21, 1.81]
**Subtotal (95% CI)**			**889**			**860**	**45.5%**	**1.51 [1.04, 1.98]**
Heterogeneity: Tau² = 0.90; Chi² = 279.61, df = 16 (P < 0.00001); I² = 94%								
Test for overall effect: Z = 6.30 (P < 0.00001)								
**1.7.2 Theory course**								
Chen LM et al. 2020	89.79	10.52	100	72.94	6.18	100	2.8%	1.95 [1.61, 2.28]
Chu L et al. 2021	83.95	7.42	87	79.37	10.16	89	2.8%	0.51 [0.21, 0.81]
Deng HX et al. 2019	81.39	1.65	57	71.31	1.8	55	2.4%	5.80 [4.94, 6.66]
Duan LH et al. 2021	76.56	8.54	55	71.69	7.82	52	2.8%	0.59 [0.20, 0.98]
Guo F et al. 2018	88.78	5.65	118	79.12	4.27	120	2.8%	1.93 [1.62, 2.23]
Li L et al. 2021	73.92	6.99	81	71.86	8.44	74	2.8%	0.27 [-0.05, 0.58]
Li S et al. 2022	75.79	10.15	57	69.64	16.79	28	2.8%	0.48 [0.02, 0.94]
Liu XY et al 2022	83.9	11.38	518	81.1	13.72	532	2.9%	0.22 [0.10, 0.34]
Liu YD et al. 2020	81.33	9.73	36	73.21	9.98	36	2.7%	0.82 [0.33, 1.30]
Ma B et al. 2019	88.1	4.7	25	63.3	11.1	26	2.5%	2.84 [2.05, 3.64]
Parhatti R et al. 2018	89.83	5.6	32	78.65	5.49	32	2.6%	1.99 [1.39, 2.60]
Shen XJ et al. 2016	79.91	8.89	32	74.5	9.54	34	2.7%	0.58 [0.09, 1.07]
Shen XJ et al. 2017	79.99	8.31	68	76.11	9.68	66	2.8%	0.43 [0.09, 0.77]
Sun YR et al. 2020	46.03	2.31	55	34.85	2.76	55	2.6%	4.36 [3.67, 5.06]
Wan YX et al. 2020	87.69	11.57	32	78.42	17.21	32	2.7%	0.62 [0.12, 1.13]
Xu YJ et al. 2019	76.1	13.19	98	66.02	17.35	63	2.8%	0.67 [0.35, 1.00]
Yang XH et al. 2018	89.23	5.76	116	78.93	7.89	119	2.9%	1.48 [1.19, 1.77]
Zhang DD et al. 2021	90.46	5.31	30	74.98	8.36	30	2.6%	2.18 [1.53, 2.83]
Zhou Y et al. 2020	72.28	1.93	52	64.62	2.71	30	2.6%	3.38 [2.69, 4.08]
Zhou Y et al. 2021	88.27	2.73	30	71.23	9.87	48	2.7%	2.12 [1.55, 2.69]
**Subtotal (95% CI)**			**1679**			**1621**	**54.5%**	**1.61 [1.15, 2.06]**
Heterogeneity: Tau² = 1.02; Chi² = 589.78, df = 19 (P < 0.00001); I² = 97%								
Test for overall effect: Z = 6.91 (P < 0.00001)								
**Total (95% CI)**			**2568**			**2481**	**100.0%**	**1.56 [1.24, 1.88]**
Heterogeneity: Tau² = 0.92; Chi² = 891.82, df = 36 (P < 0.00001); I² = 96%								
Test for overall effect: Z = 9.51 (P < 0.00001)								
Test for subgroup differences: Chi² = 0.08, df = 1 (P = 0.78), I² = 0%								

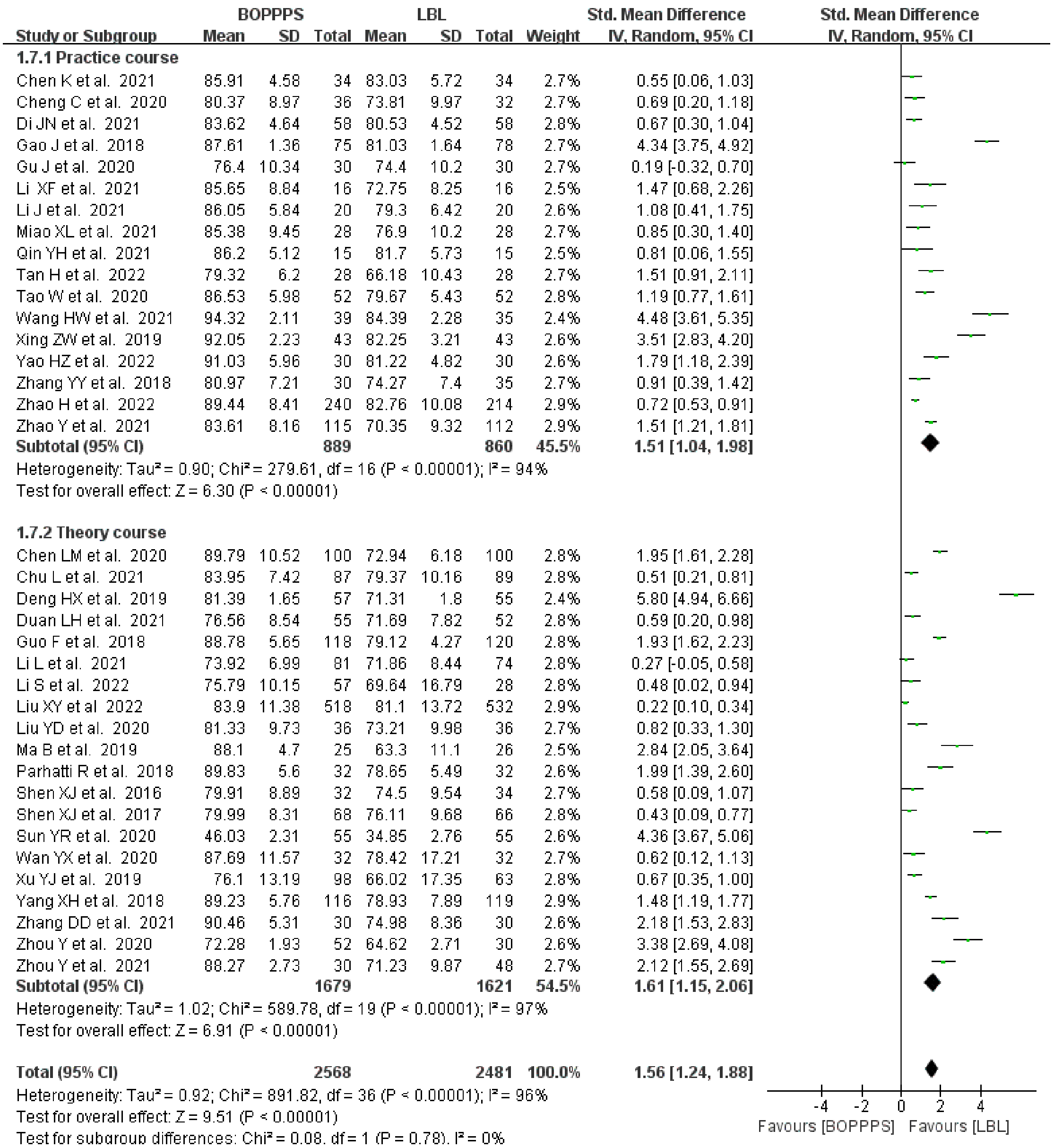

Appendix 5. Forest plot of subgroup analyses of final knowledge examination score

5.5 Subgroup analyses of course contents

Study or Subgroup	BOPPPS Mean	BOPPPS SD	BOPPPS Total	LBL Mean	LBL SD	LBL Total	Weight	Std. Mean Difference IV, Random, 95% CI
**1.8.1 Anesthesiology**								
Miao XL et al. 2021	85.38	9.45	28	76.9	10.2	28	2.7%	0.85 [0.30, 1.40]
Wan YX et al. 2020	87.69	11.57	32	78.42	17.21	32	2.7%	0.62 [0.12, 1.13]
**Subtotal (95% CI)**			**60**			**60**	**5.4%**	**0.73 [0.36, 1.10]**
Heterogeneity: Tau^2 = 0.00; Chi^2 = 0.35, df = 1 (P = 0.55); I^2 = 0%								
Test for overall effect: Z = 3.85 (P = 0.0001)								
**1.8.2 Diagnostics**								
Cheng C et al. 2020	80.37	8.97	36	73.81	9.97	74	2.8%	0.67 [0.27, 1.08]
Li XF et al. 2021	85.65	8.84	16	72.75	8.25	34	2.6%	1.50 [0.84, 2.17]
Li L et al. 2021	73.92	6.99	81	71.86	8.44	48	2.8%	0.27 [-0.09, 0.63]
Shen XJ et al. 2016	79.91	8.89	32	74.5	9.54	32	2.7%	0.58 [0.08, 1.08]
Zhou Y et al. 2020	72.28	1.93	52	64.62	2.71	16	2.4%	3.55 [2.72, 4.38]
**Subtotal (95% CI)**			**217**			**204**	**13.4%**	**1.25 [0.41, 2.10]**
Heterogeneity: Tau^2 = 0.85; Chi^2 = 55.74, df = 4 (P < 0.00001); I^2 = 93%								
Test for overall effect: Z = 2.90 (P = 0.004)								
**1.8.3 Internal medicine**								
Di JN et al. 2021	83.62	4.64	58	80.53	4.52	78	2.8%	0.67 [0.32, 1.02]
Gao J et al. 2018	87.61	1.36	75	81.03	1.64	20	2.4%	4.59 [3.76, 5.42]
Li J et al. 2021	86.05	5.84	20	79.3	6.42	66	2.7%	1.06 [0.54, 1.59]
Qin YH et al. 2021	86.2	5.12	15	81.7	5.73	15	2.5%	0.81 [0.06, 1.55]
Shen XJ et al. 2017	79.99	8.31	68	76.11	9.68	35	2.8%	0.44 [0.03, 0.85]
Wang HW et al. 2021	94.32	2.11	39	84.39	2.28	43	2.4%	4.47 [3.65, 5.29]
Xing ZW et al. 2019	92.05	2.23	43	82.25	3.21	58	2.6%	3.43 [2.81, 4.05]
Zhao H et al. 2022	89.44	8.41	240	82.76	10.08	214	2.9%	0.72 [0.53, 0.91]
Zhou Y et al. 2021	88.27	2.73	30	71.23	9.87	30	2.6%	2.32 [1.66, 2.99]
**Subtotal (95% CI)**			**588**			**559**	**23.9%**	**2.01 [1.19, 2.84]**
Heterogeneity: Tau^2 = 1.50; Chi^2 = 230.39, df = 8 (P < 0.00001); I^2 = 97%								
Test for overall effect: Z = 4.77 (P < 0.00001)								
**1.8.4 Obstetrics & gynecology**								
Yang XH et al. 2018	89.23	5.76	116	78.93	7.89	119	2.9%	1.48 [1.19, 1.77]
Zhang DD et al. 2021	90.46	5.31	30	74.98	8.36	30	2.6%	2.18 [1.53, 2.83]
Zhang YY et al. 2018	80.97	7.21	30	74.27	7.4	35	2.7%	0.91 [0.39, 1.42]
**Subtotal (95% CI)**			**176**			**184**	**8.2%**	**1.49 [0.91, 2.08]**
Heterogeneity: Tau^2 = 0.21; Chi^2 = 9.28, df = 2 (P = 0.010); I^2 = 78%								
Test for overall effect: Z = 4.99 (P < 0.00001)								
**1.8.5 Surgery**								
Duan LH et al. 2021	76.56	8.54	55	71.69	7.82	112	2.8%	0.60 [0.27, 0.93]
Gu J et al. 2020	76.4	10.34	30	74.4	10.2	52	2.8%	0.19 [-0.26, 0.64]
Liu YD et al. 2020	81.33	9.73	36	73.21	9.98	30	2.7%	0.82 [0.31, 1.32]
Parhatti R et al. 2018	89.83	5.6	32	78.65	5.49	52	2.7%	2.00 [1.46, 2.54]
Tao W et al. 2020	86.53	5.98	52	79.67	5.43	32	2.7%	1.18 [0.70, 1.65]
Zhao Y et al. 2021	83.61	8.16	115	70.35	9.32	36	2.8%	1.56 [1.15, 1.98]
**Subtotal (95% CI)**			**320**			**314**	**16.6%**	**1.05 [0.54, 1.55]**
Heterogeneity: Tau^2 = 0.35; Chi^2 = 39.27, df = 5 (P < 0.00001); I^2 = 87%								
Test for overall effect: Z = 4.06 (P < 0.0001)								
**1.8.6 Traditional chinese medicine**								
Chen K et al. 2021	85.91	4.58	34	83.03	5.72	34	2.7%	0.55 [0.06, 1.03]
Li S et al. 2022	75.79	10.15	57	69.64	16.79	28	2.8%	0.48 [0.02, 0.94]
Xu YJ et al. 2019	76.1	13.19	98	66.02	17.35	63	2.8%	0.67 [0.35, 1.00]
Yao HZ et al. 2022	91.03	5.96	30	81.22	4.82	30	2.6%	1.79 [1.18, 2.39]
**Subtotal (95% CI)**			**219**			**155**	**11.0%**	**0.83 [0.35, 1.32]**
Heterogeneity: Tau^2 = 0.19; Chi^2 = 13.51, df = 3 (P = 0.004); I^2 = 78%								
Test for overall effect: Z = 3.38 (P = 0.0007)								
**1.8.7 Pathology**								
Chen LM et al. 2020	89.79	10.52	100	72.94	6.18	100	2.8%	1.95 [1.61, 2.28]
Sun YR et al. 2020	46.03	2.31	55	34.85	2.76	55	2.6%	4.36 [3.67, 5.06]
**Subtotal (95% CI)**			**155**			**155**	**5.4%**	**3.13 [0.77, 5.50]**
Heterogeneity: Tau^2 = 2.84; Chi^2 = 37.50, df = 1 (P < 0.00001); I^2 = 97%								
Test for overall effect: Z = 2.59 (P = 0.009)								
**1.8.8 Emergency medicine**								
Guo F et al. 2018	88.78	5.65	118	79.12	4.27	120	2.9%	1.93 [1.62, 2.23]
**Subtotal (95% CI)**			**118**			**120**	**2.9%**	**1.93 [1.62, 2.23]**
Heterogeneity: Not applicable								
Test for overall effect: Z = 12.24 (P < 0.00001)								
**1.8.9 Evidence-based medicine**								
Ma B et al. 2019	88.1	4.7	25	63.3	11.1	26	2.5%	2.84 [2.05, 3.64]
**Subtotal (95% CI)**			**25**			**26**	**2.5%**	**2.84 [2.05, 3.64]**
Heterogeneity: Not applicable								
Test for overall effect: Z = 7.02 (P < 0.00001)								
**1.8.10 General Medicine**								
Deng HX et al. 2019	81.39	1.65	57	71.31	1.8	55	2.4%	5.80 [4.94, 6.66]
**Subtotal (95% CI)**			**57**			**55**	**2.4%**	**5.80 [4.94, 6.66]**
Heterogeneity: Not applicable								
Test for overall effect: Z = 13.26 (P < 0.00001)								
**1.8.11 Pediatrics**								
Tan H et al. 2022	79.32	6.2	28	66.18	10.43	28	2.7%	1.51 [0.91, 2.11]
**Subtotal (95% CI)**			**28**			**28**	**2.7%**	**1.51 [0.91, 2.11]**
Heterogeneity: Not applicable								
Test for overall effect: Z = 4.94 (P < 0.00001)								
**1.8.12 Human anatomy**								
Chu L et al. 2021	83.95	7.42	87	79.37	10.16	89	2.9%	0.51 [0.21, 0.81]
**Subtotal (95% CI)**			**87**			**89**	**2.9%**	**0.51 [0.21, 0.81]**
Heterogeneity: Not applicable								
Test for overall effect: Z = 3.34 (P = 0.0008)								
**1.8.13 Physiology**								
Liu XY et al 2022	83.9	11.38	518	81.1	13.72	532	2.9%	0.22 [0.10, 0.34]
**Subtotal (95% CI)**			**518**			**532**	**2.9%**	**0.22 [0.10, 0.34]**
Heterogeneity: Not applicable								
Test for overall effect: Z = 3.58 (P = 0.0003)								
**Total (95% CI)**			**2568**			**2481**	**100.0%**	**1.57 [1.25, 1.88]**
Heterogeneity: Tau^2 = 0.88; Chi^2 = 836.18, df = 36 (P < 0.00001); I^2 = 96%								
Test for overall effect: Z = 9.74 (P < 0.00001)								
Test for subgroup differences: Chi^2 = 317.28, df = 12 (P < 0.00001), I^2 = 96.2%								

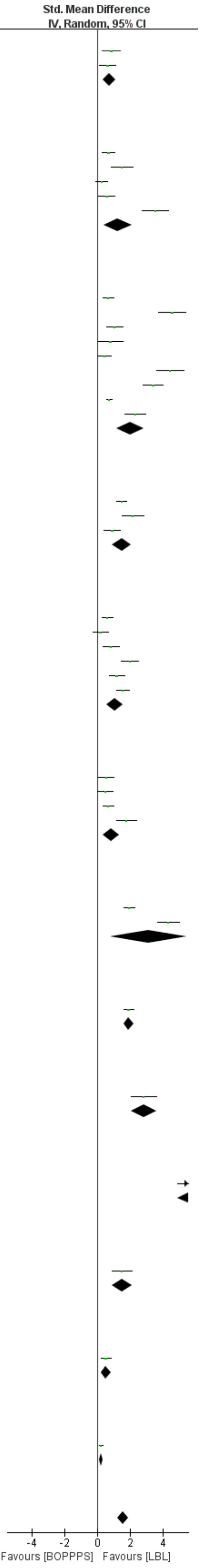

Appendix 6. Forest plot of subgroup analyses of comprehensive ability score

6.1 Subgroup analyses of training levels

Study or Subgroup	BOPPPS Mean	BOPPPS SD	BOPPPS Total	LBL Mean	LBL SD	LBL Total	Weight	Std. Mean Difference IV, Random, 95% CI
**2.1.1 Graduates**								
Chen K et al. 2021	84.96	5.2	34	81.39	5.56	34	5.3%	0.66 [0.17, 1.14]
Gu J et al. 2020	76.04	9.02	30	71.12	10.73	30	5.2%	0.49 [-0.02, 1.00]
Li XF et al. 2021	82.57	8.15	16	73.55	8.14	16	4.7%	1.08 [0.33, 1.83]
Miao XL et al. 2021	61.9	4.57	28	54.6	4.87	28	5.0%	1.52 [0.92, 2.12]
Zhao H et al. 2022	96.01	1.62	240	95.42	2.35	214	5.7%	0.29 [0.11, 0.48]
**Subtotal (95% CI)**			**348**			**322**	**26.0%**	**0.75 [0.31, 1.19]**
Heterogeneity: Tau² = 0.18; Chi² = 18.39, df = 4 (P = 0.001); I² = 78%								
Test for overall effect: Z = 3.34 (P = 0.0008)								
**2.1.2 Undergraduates**								
Chen LM et al. 2020	90.24	7.58	100	76.84	10.35	100	5.6%	1.47 [1.16, 1.78]
Cheng C et al. 2020	81.88	7.29	36	75.06	8.87	32	5.3%	0.84 [0.34, 1.33]
Chu L et al. 2021	81.56	9.89	87	76.8	12.44	89	5.6%	0.42 [0.12, 0.72]
Di JN et al. 2021	87.57	4.1	58	78.62	4.66	58	5.4%	2.03 [1.58, 2.48]
Duan LH et al. 2021	78.93	8.01	55	73.59	8.06	52	5.5%	0.66 [0.27, 1.05]
Li J et al. 2018	82.45	5.27	108	77.48	7	109	5.6%	0.80 [0.52, 1.08]
Li J et al. 2021	82.6	6.94	20	76.15	6.97	20	4.9%	0.91 [0.25, 1.56]
Li L et al. 2021	75.21	9.16	81	74.98	6.35	74	5.6%	0.03 [-0.29, 0.34]
Li SF et al. 2017	92.8	2.5	66	86	4.7	69	5.4%	1.79 [1.38, 2.19]
Liu Q et al. 2019	84.29	5.49	30	80.3	6.27	30	5.2%	0.67 [0.15, 1.19]
Tan H et al. 2022	97.79	4.36	28	89.46	9.75	28	5.1%	1.09 [0.52, 1.65]
Xing ZW et al. 2019	93.24	1.9	43	83.95	2.75	43	4.7%	3.90 [3.16, 4.63]
Yang Y et al. 2021	83.43	2.93	51	66.67	6.65	48	5.0%	3.27 [2.66, 3.88]
Yao HZ et al. 2022	90.63	5.91	30	80.82	4.75	30	5.0%	1.81 [1.20, 2.41]
**Subtotal (95% CI)**			**793**			**782**	**74.0%**	**1.37 [0.91, 1.84]**
Heterogeneity: Tau² = 0.72; Chi² = 220.37, df = 13 (P < 0.00001); I² = 94%								
Test for overall effect: Z = 5.80 (P < 0.00001)								
**Total (95% CI)**			**1141**			**1104**	**100.0%**	**1.22 [0.85, 1.59]**
Heterogeneity: Tau² = 0.60; Chi² = 277.40, df = 18 (P < 0.00001); I² = 94%								
Test for overall effect: Z = 6.49 (P < 0.00001)								
Test for subgroup differences: Chi² = 3.64, df = 1 (P = 0.06), I² = 72.5%								

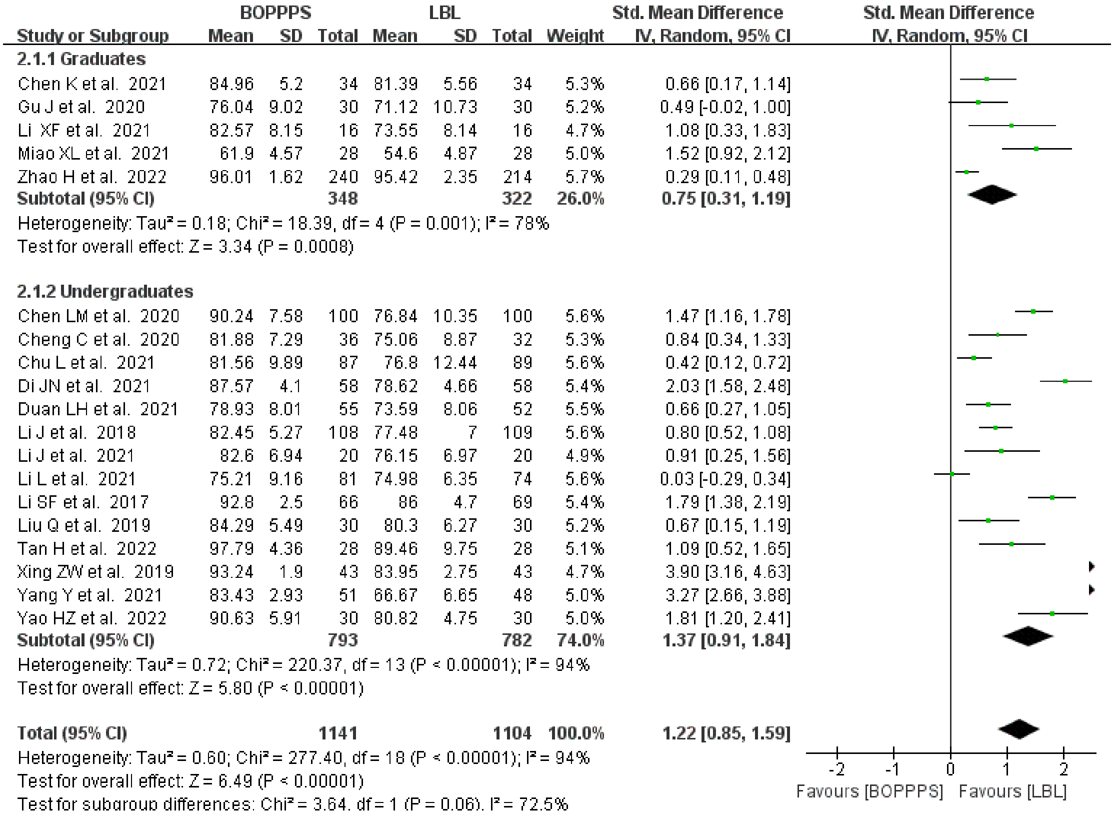

Appendix 6. Forest plot of subgroup analyses of comprehensive ability score

6.2 Subgroup analyses of course type

	BOPPPS			LBL				Std. Mean Difference
Study or Subgroup	Mean	SD	Total	Mean	SD	Total	Weight	IV, Random, 95% CI
**2.2.1 Theory**								
Chen LM et al. 2020	90.24	7.58	100	76.84	10.35	100	5.6%	1.47 [1.16, 1.78]
Chu L et al. 2021	81.56	9.89	87	76.8	12.44	89	5.6%	0.42 [0.12, 0.72]
Duan LH et al. 2021	78.93	8.01	55	73.59	8.06	52	5.5%	0.66 [0.27, 1.05]
Li L et al. 2021	75.21	9.16	81	74.98	6.35	74	5.6%	0.03 [-0.29, 0.34]
Yang Y et al. 2021	83.43	2.93	51	66.67	6.65	48	5.0%	3.27 [2.66, 3.88]
**Subtotal (95% CI)**			**374**			**363**	**27.3%**	**1.14 [0.30, 1.98]**
Heterogeneity: Tau² = 0.88; Chi² = 110.43, df = 4 (P < 0.00001); I² = 96%								
Test for overall effect: Z = 2.65 (P = 0.008)								
**2.2.2 Practice**								
Chen K et al. 2021	84.96	5.2	34	81.39	5.56	34	5.3%	0.66 [0.17, 1.14]
Cheng C et al. 2020	81.88	7.29	36	75.06	8.87	32	5.3%	0.84 [0.34, 1.33]
Di JN et al. 2021	87.57	4.1	58	78.62	4.66	58	5.4%	2.03 [1.58, 2.48]
Gu J et al. 2020	76.04	9.02	30	71.12	10.73	30	5.2%	0.49 [-0.02, 1.00]
Li XF et al. 2021	82.57	8.15	16	73.55	8.14	16	4.7%	1.08 [0.33, 1.83]
Li J et al. 2018	82.45	5.27	108	77.48	7	109	5.6%	0.80 [0.52, 1.08]
Li J et al. 2021	82.6	6.94	20	76.15	6.97	20	4.9%	0.91 [0.25, 1.56]
Li SF et al. 2017	92.8	2.5	66	86	4.7	69	5.4%	1.79 [1.38, 2.19]
Liu Q et al. 2019	84.29	5.49	30	80.3	6.27	30	5.2%	0.67 [0.15, 1.19]
Miao XL et al. 2021	61.9	4.57	28	54.6	4.87	28	5.0%	1.52 [0.92, 2.12]
Tan H et al. 2022	97.79	4.36	28	89.46	9.75	28	5.1%	1.09 [0.52, 1.65]
Xing ZW et al. 2019	93.24	1.9	43	83.95	2.75	43	4.7%	3.90 [3.16, 4.63]
Yao HZ et al. 2022	90.63	5.91	30	80.82	4.75	30	5.0%	1.81 [1.20, 2.41]
Zhao H et al. 2022	96.01	1.62	240	95.42	2.35	214	5.7%	0.29 [0.11, 0.48]
**Subtotal (95% CI)**			**767**			**741**	**72.7%**	**1.25 [0.83, 1.67]**
Heterogeneity: Tau² = 0.57; Chi² = 166.37, df = 13 (P < 0.00001); I² = 92%								
Test for overall effect: Z = 5.82 (P < 0.00001)								
**Total (95% CI)**			**1141**			**1104**	**100.0%**	**1.22 [0.85, 1.59]**
Heterogeneity: Tau² = 0.60; Chi² = 277.40, df = 18 (P < 0.00001); I² = 94%								
Test for overall effect: Z = 6.49 (P < 0.00001)								
Test for subgroup differences: Chi² = 0.05, df = 1 (P = 0.82), I² = 0%								

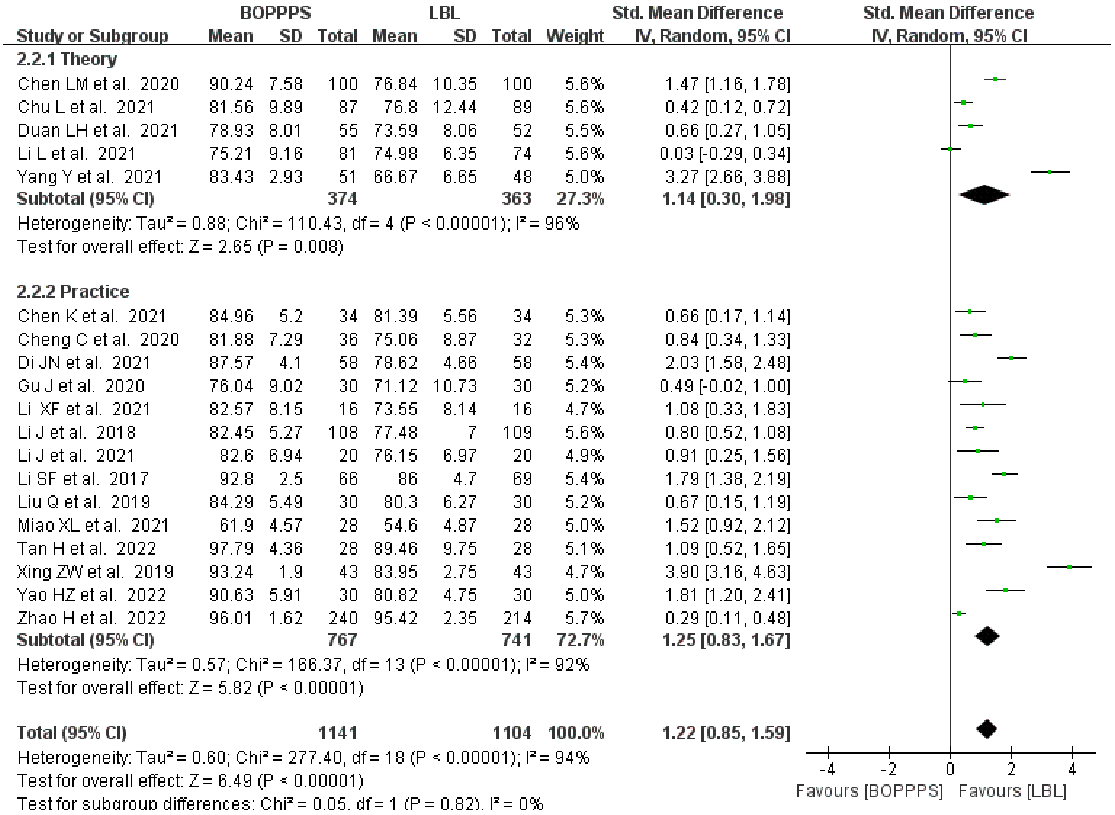

Appendix 6 Forest plot of subgroup analyses of comprehensive ability score

6.3 Subgroup analyses of course contents

Study or Subgroup	BOPPPS Mean	BOPPPS SD	BOPPPS Total	LBL Mean	LBL SD	LBL Total	Weight	Std. Mean Difference IV, Random, 95% CI
**2.3.1 Anesthesiology**								
Miao XL et al. 2021	61.9	4.57	28	54.6	4.87	28	5.0%	1.52 [0.92, 2.12]
**Subtotal (95% CI)**			**28**			**28**	**5.0%**	**1.52 [0.92, 2.12]**
Heterogeneity: Not applicable								
Test for overall effect: Z = 4.98 (P < 0.00001)								
**2.3.2 Diagnostics**								
Cheng C et al. 2020	81.88	7.29	36	75.06	8.87	74	5.4%	0.81 [0.39, 1.22]
Li XF et al. 2021	82.57	8.15	16	73.55	8.14	32	4.9%	1.09 [0.45, 1.73]
Li L et al. 2021	75.21	9.16	81	74.98	6.35	16	5.2%	0.03 [-0.51, 0.56]
**Subtotal (95% CI)**			**133**			**122**	**15.5%**	**0.63 [0.04, 1.22]**
Heterogeneity: Tau² = 0.20; Chi² = 7.55, df = 2 (P = 0.02); I² = 74%								
Test for overall effect: Z = 2.11 (P = 0.04)								
**2.3.3 Human anatomy**								
Chu L et al. 2021	81.56	9.89	87	76.8	12.44	89	5.6%	0.42 [0.12, 0.72]
Yang Y et al. 2021	83.43	2.93	51	66.67	6.65	48	5.0%	3.27 [2.66, 3.88]
**Subtotal (95% CI)**			**138**			**137**	**10.6%**	**1.83 [-0.96, 4.63]**
Heterogeneity: Tau² = 4.00; Chi² = 67.69, df = 1 (P < 0.00001); I² = 99%								
Test for overall effect: Z = 1.29 (P = 0.20)								
**2.3.4 Internal medicine**								
Di JN et al. 2021	87.57	4.1	58	78.62	4.66	20	5.0%	2.09 [1.48, 2.70]
Li J et al. 2021	82.6	6.94	20	76.15	6.97	43	5.1%	0.92 [0.36, 1.47]
Xing ZW et al. 2019	93.24	1.9	43	83.95	2.75	58	4.9%	3.80 [3.14, 4.46]
Zhao H et al. 2022	96.01	1.62	240	95.42	2.35	214	5.8%	0.29 [0.11, 0.48]
**Subtotal (95% CI)**			**361**			**335**	**20.8%**	**1.75 [0.27, 3.24]**
Heterogeneity: Tau² = 2.22; Chi² = 122.48, df = 3 (P < 0.00001); I² = 98%								
Test for overall effect: Z = 2.31 (P = 0.02)								
**2.3.5 Obstetrics & gynecology**								
Li J et al. 2018	82.45	5.27	108	77.48	7	109	5.6%	0.80 [0.52, 1.08]
**Subtotal (95% CI)**			**108**			**109**	**5.6%**	**0.80 [0.52, 1.08]**
Heterogeneity: Not applicable								
Test for overall effect: Z = 5.66 (P < 0.00001)								
**2.3.6 Pediatrics**								
Li SF et al. 2017	92.8	2.5	66	86	4.7	69	5.5%	1.79 [1.38, 2.19]
Tan H et al. 2022	97.79	4.36	28	89.46	9.75	28	5.1%	1.09 [0.52, 1.65]
**Subtotal (95% CI)**			**94**			**97**	**10.6%**	**1.47 [0.78, 2.15]**
Heterogeneity: Tau² = 0.18; Chi² = 3.90, df = 1 (P = 0.05); I² = 74%								
Test for overall effect: Z = 4.22 (P < 0.0001)								
**2.3.7 Surgery**								
Duan LH et al. 2021	78.93	8.01	55	73.59	8.06	52	5.5%	0.66 [0.27, 1.05]
Gu J et al. 2020	76.04	9.02	30	71.12	10.73	30	5.2%	0.49 [-0.02, 1.00]
**Subtotal (95% CI)**			**85**			**82**	**10.7%**	**0.60 [0.29, 0.91]**
Heterogeneity: Tau² = 0.00; Chi² = 0.27, df = 1 (P = 0.61); I² = 0%								
Test for overall effect: Z = 3.77 (P = 0.0002)								
**2.3.8 Traditional chinese medicine**								
Chen K et al. 2021	84.96	5.2	34	81.39	5.56	34	5.3%	0.66 [0.17, 1.14]
Liu Q et al. 2019	84.29	5.49	30	80.3	6.27	30	5.2%	0.67 [0.15, 1.19]
Yao HZ et al. 2022	90.63	5.91	30	80.82	4.75	30	5.0%	1.81 [1.20, 2.41]
**Subtotal (95% CI)**			**94**			**94**	**15.5%**	**1.02 [0.33, 1.72]**
Heterogeneity: Tau² = 0.31; Chi² = 10.17, df = 2 (P = 0.006); I² = 80%								
Test for overall effect: Z = 2.87 (P = 0.004)								
**2.3.9 Pathology**								
Chen LM et al. 2020	90.24	7.58	100	76.84	10.35	100	5.6%	1.47 [1.16, 1.78]
**Subtotal (95% CI)**			**100**			**100**	**5.6%**	**1.47 [1.16, 1.78]**
Heterogeneity: Not applicable								
Test for overall effect: Z = 9.21 (P < 0.00001)								
**Total (95% CI)**			**1141**			**1104**	**100.0%**	**1.22 [0.86, 1.58]**
Heterogeneity: Tau² = 0.58; Chi² = 258.30, df = 18 (P < 0.00001); I² = 93%								
Test for overall effect: Z = 6.60 (P < 0.00001)								
Test for subgroup differences: Chi² = 24.75, df = 8 (P = 0.002), I² = 67.7%								

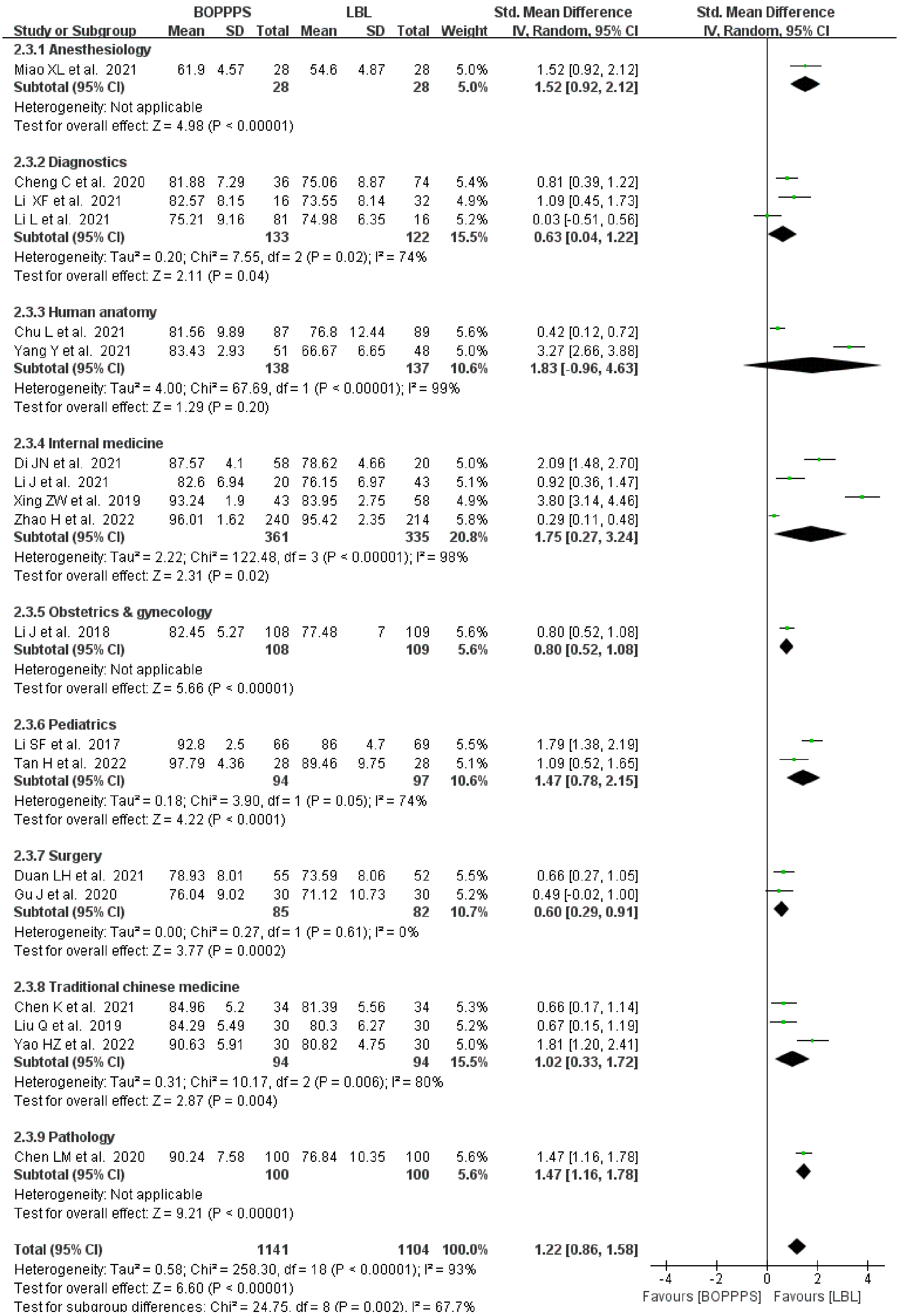

Appendix 7. Sensitive analysis of all the studies using Metaninf function in Stata.

(A) Sensitive analysis of knowledge examination scores; (B) Sensitive analysis of comprehensive ability scores.

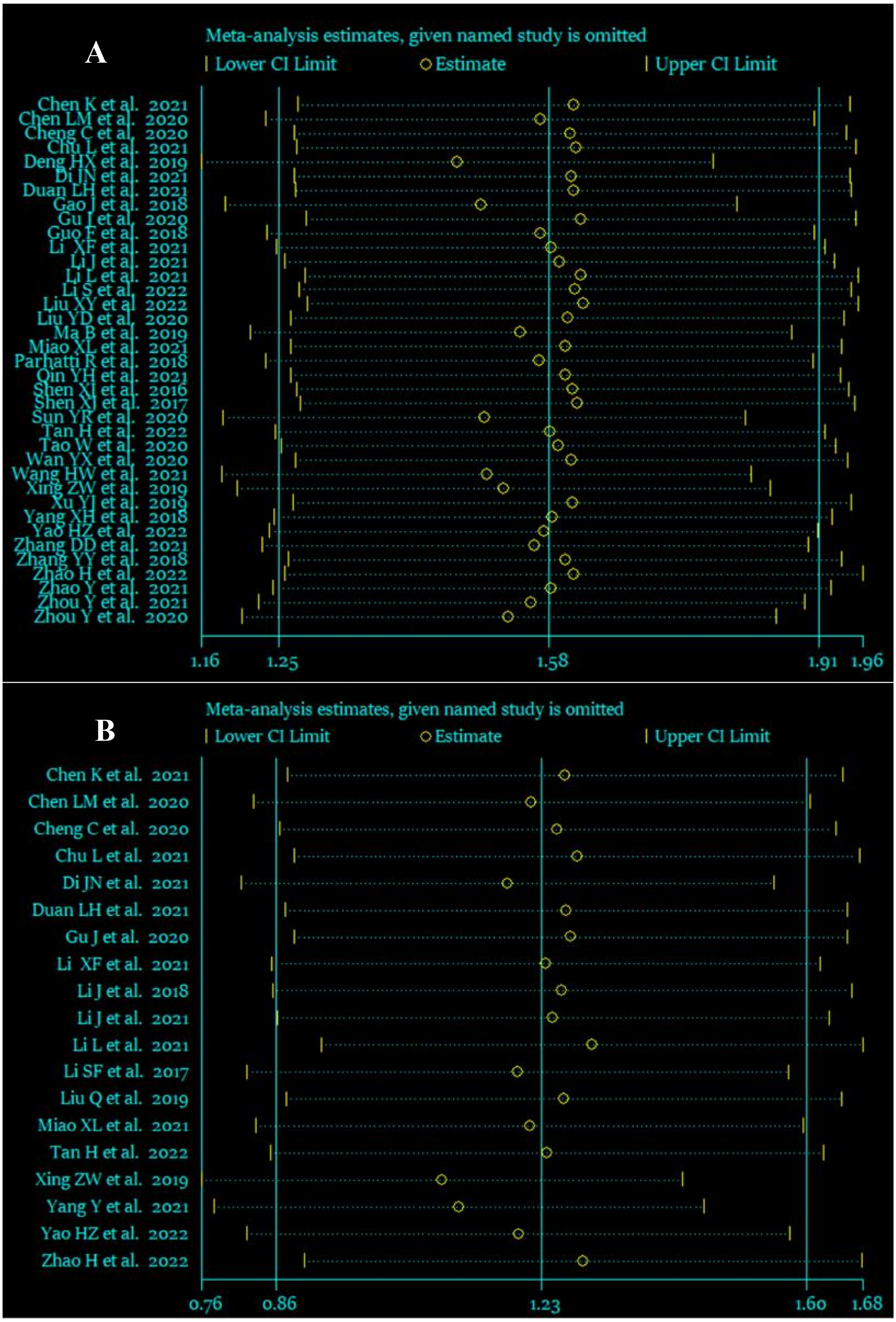

Supplement: Supplementary file 1 [file Data_Sheet_1.PDF]
